# Lignin beyond the *status quo*: recent and emerging composite applications

**DOI:** 10.1039/d3gc03154c

**Published:** 2023-12-01

**Authors:** Mahyar Fazeli, Sritama Mukherjee, Hossein Baniasadi, Roozbeh Abidnejad, Muhammad Mujtaba, Juha Lipponen, Jukka Seppälä, Orlando J. Rojas

**Affiliations:** a Department of Bioproducts and Biosystems, School of Chemical Engineering, Aalto University FI-00076 Aalto Finland mahyar.fazeli@aalto.fi; b Division of Fiber and Polymer Technology, CBH, KTH Royal Institute of Technology Teknikringen 56-58 SE-100 44 Stockholm Sweden; c Polymer Technology, School of Chemical Engineering, Aalto University Espoo Finland; d VTT Technical Research Centre of Finland Ltd P.O. Box 1000 Espoo FI-02044 Finland; e Bioproducts Institute, Department of Chemical & Biological Engineering, Department of Chemistry, Department of Wood Science, 2360 East Mall, The University of British Columbia Vancouver BC V6T 1Z3 Canada orlando.rojas@ubc.ca

## Abstract

The demand for biodegradable materials across various industries has recently surged due to environmental concerns and the need for the adoption of renewable materials. In this context, lignin has emerged as a promising alternative, garnering significant attention as a biogenic resource that endows functional properties. This is primarily ascribed to its remarkable origin and structure that explains lignin's capacity to bind other molecules, reinforce composites, act as an antioxidant, and endow antimicrobial effects. This review summarizes recent advances in lignin-based composites, with particular emphasis on innovative methods for modifying lignin into micro and nanostructures and evaluating their functional contribution. Indeed, lignin-based composites can be tailored to have superior physicomechanical characteristics, biodegradability, and surface properties, thereby making them suitable for applications beyond the typical, for instance, in ecofriendly adhesives and advanced barrier technologies. Herein, we provide a comprehensive overview of the latest progress in the field of lignin utilization in emerging composite materials.

## Abstract 2

Potential applications of lignin.

## Graphical abstract

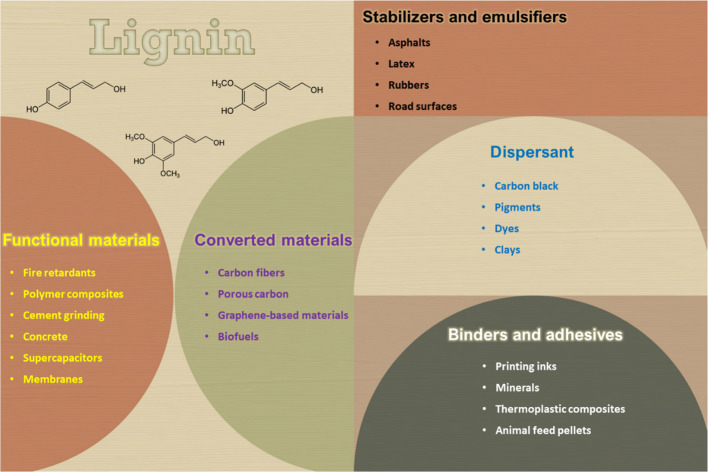

## 1. Introduction

The utilization of biodegradable materials has gained significant popularity in the industrial sector for property development, material and energy integration, recyclability, and product life. With the concerns about the environmental impact of non-renewable resources, access to raw materials, and product end-of-life, there is a compelling need to explore new alternatives in the chemical industries.^1,2^ Lignin, a macromolecule present in wood and other plants, emerges as a promising sustainable biomass resource to tackle these challenges. Although lignin has traditionally been used for energy co-generation, it is now well recognized as a viable substitute for traditional components in various applications.^3–5^

From the chemical perspective, lignin is one of the most abundant polymeric materials of biological origin, comprising a complex polyaromatic structure.^6,7^ It demonstrates considerable potential in green technologies.^8^ As a crucial component of plant cell walls, lignin acts as a binder between cellulose and hemicelluloses.^9^ Isolation of lignin from agricultural and wood residues involves known processes, with precipitation methods as an effective means to separate lignin from industrial liquors produced during the delignification of biomass, the so-called black liquors.^10^ The presence of different polar functional groups in lignin enables chemical bonding with other molecules. Moreover, lignin possesses excellent reinforcing properties, making it suitable as a filler in composites. The utilization of lignin-based materials and composites has already become a significant area of focus, and increased research and development efforts have taken place over the years.^5,11–13^

To gain a comprehensive understanding of lignin's significance in various applications, including composites, it is essential to recognize its complex nature as a polymer composed of three phenylpropanoid monomers: coniferyl alcohol, coumaryl alcohol, and sinapyl alcohol. Lignin exists in a three-dimensional structure that is non-uniform throughout, with each monomer unit offering multiple sites for polymerization, allowing for a diverse range of connections between units. The composition of lignin, which varies among plant species, plays a crucial role. Softwood, for instance, typically contains approximately 45–50% cellulose, 25–35% hemicellulose, and 25–35% lignin. Moreover, the monomer composition of lignin is influenced by the specific plant species.^14^

Lignin exhibits considerable variability in both production methods and chemical composition, primarily due to the industrial extraction techniques used on lignocellulosic biomass. This variability sets it apart from cellulose. Currently, several methods are utilized to extract lignin from lignocellulosic biomass, with many integrated into pulping processes. These pulping processes can be broadly classified into three main subtypes: chemical, semi-mechanical, and mechanical pulping. In certain cases, these methods are combined to achieve desired outcomes. Mechanical pulping, commonly employed in low-cost paper industries, predominantly involves nonresinous softwoods and certain hardwoods, resulting in a yellowish form of lignin. Mechanical pulping relies solely on water or steam without the use of chemical additives. Conversely, hardwoods, which often yield lower-quality pulp, undergo a combination of chemical and mechanical pulping techniques. Chemical pulping, on the other hand, yields various forms of technical lignin, including kraft lignin, soda lignin, and lignosulphonates, which are extensively utilized in the paper industry. However, lignin forms such as hydrolysis lignin, ionic liquid lignin, and organosolv lignin are generated in smaller quantities.^15,16^

**Fig. 1:**
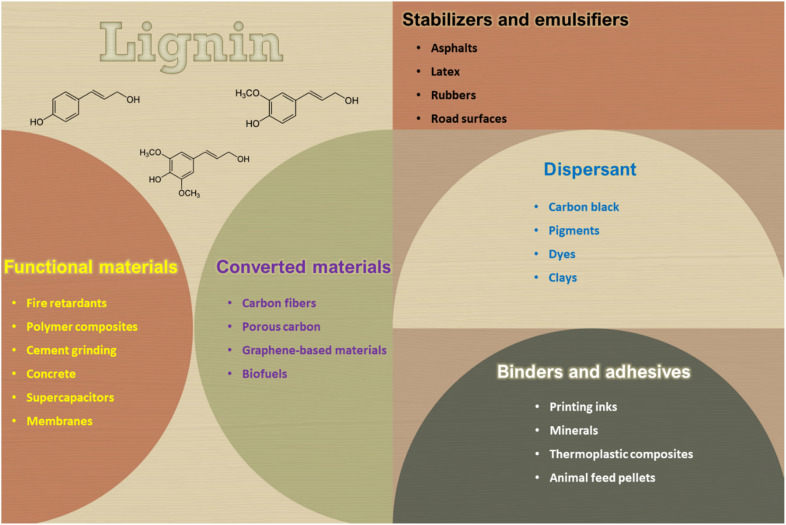
Potential applications of lignin.

The field of lignin-based materials and composites has garnered attention from both industry and academia, Fig. 1. Conventional methods can be employed to produce lignin derivatives with micro- and nanostructural characteristics that enhance performance and offer practical avenues for composite modification. Customizable lignin particles can be developed to improve given functions.^13,17–20^ Lignin's specific network structure, combined with its diverse chemical groups, contributes to its functional properties, including biodegradability, reinforcement characteristics, ultraviolet (UV) absorption, multiphase stabilization, and antibiotic and antimicrobial capacity, among others.^11,21–23^

Incorporating lignin into composites enhances strength, stiffness, and toughness. Lignin-derived composites show significant potential in improving thermal and barrier properties, making them suitable for a wide range of uses, from the construction and automotive industries to packaging and functional coatings.^24–26^ Lignin's ability to form strong bonds with other molecules makes it suitable as a component of adhesive formulations. Furthermore, lignin-based composites exhibit unique rheological properties that enable processing through various techniques such as extrusion, compression molding, and 3D printing.^27^ Modifying the lignin structure allows for tailoring the surface properties of lignin-based composites, resulting in improved water resistance, biodegradability, and surface energy.^28^ These properties make composites attractive for advanced applications, including those in the biomedical field, drug delivery, and tissue engineering.^29^

This review presents a comprehensive overview of recent advancements in the emerging field of lignin-based sustainable composites. In particular, we explore the latest developments, including the utilization of lignin micro- and nanostructures, and their potential to enhance the performance of functional composites in various applications. To underscore the significance of this review, it is valuable to compare it with previous works on the subject. For example, Li *et al.*^30^ focused exclusively on the importance of lignin in phenolic resins and related aspects. Furthermore, Ma *et al.*^31^ highlighted the critical role of lignin in energy storage and electrical applications. There are other notable examples as well, such as Parvathy *et al.*,^32^ who primarily concentrated on lignin at the nanoscale and its applications, and Collins *et al.*,^33^ who explored the development of sustainable feedstocks for future fuels, chemicals, polymers, and fibers. In contrast, our review covers a broader range of interests and distinguishes itself by being up-to-date, comprehensive, and focusing on innovative aspects of lignin-based nanocomposites. By highlighting key advancements in this field, our review offers valuable insights into the potential of sustainable composites as alternatives to traditional materials for ecofriendly adhesives, functional fillers, and advanced barriers. Ultimately, it serves as a valuable resource for research and development in the field of sustainable composite materials.

## 2. Lignin contribution to adhesion

Adhesives play a crucial role in various sectors, such as renewable energy, building, manufacturing, transportation, packaging, and more.^34^ The adhesive market has been consistently expanding, reaching 58 billion US dollars in 2020. However, most adhesive manufacturers rely on petroleum-derived raw materials like phenol, formaldehyde, and polyether polyols. The increasing demand for these non-renewable resources not only leads to rising costs but also contributes to significant solid waste and gaseous pollutants, including carbon monoxide, sulfur dioxide, and nitrous oxides, during the processing, production, and use of fossil fuel-derived products.

With the growing wood composite industry and the rise of wood-based panels, there is a need for adhesives in the wood product manufacturing sector. Aldehyde-based adhesives such as phenol-formaldehyde (PF), urea-formaldehyde (UF), and melamine formaldehyde are commonly used in this industry due to their good adhesion, hydrophobicity, and heat resistance. However, it is important to note that phenolic resins, which are widely used in these adhesives, contain phenol and aldehyde, known for their harmful effects on human health, including the eyes, skin, and respiratory tract. Formaldehyde, in particular, has been classified as a carcinogen by the International Agency of the World Health Organization. In 2020, China's annual capacity for manufacturing, imports, and exports of WBPs was around 161 million m^3^, 3 million m^3^, and 13 million m^3^, respectively.^35^ It has been estimated that 15–17 million tons of adhesives were used in 2018–19.^36^ As the world moves towards a low-carbon economy, there is a growing emphasis on replacing petroleum-based raw materials with biomass-based chemicals. Concerns about environmental protection, the increasing price of phenol, and the need to reduce dependence on petroleum products have fueled the exploration of renewable and environmentally friendly alternatives in manufacturing processes.

**Fig. 2:**
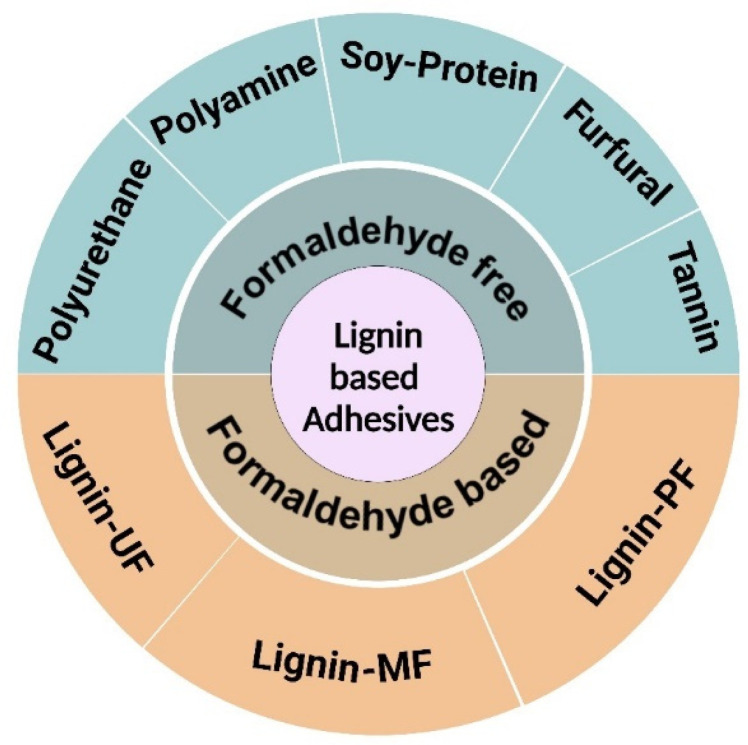
Lignin-based adhesives are divided into two categories, *i.e.*, lignin-based formaldehyde resins and lignin-based formaldehyde-free resins.

Being an inexpensive and renewable resource, lignin is a suitable replacement for phenol as it contains phenylpropane units and shares structural similarity with phenol, making it the single non-fossil source of renewable aryl compounds in nature (Fig. 2). Lignin was first used in adhesives in the late nineteenth century, and since then, lignin-based adhesives technology, particularly lignin-based phenolic formaldehyde adhesives, continued to advance and develop. Lignin has the potential to partially substitute the phenol or formaldehyde, thanks to the presence of phenolic and aliphatic hydroxyls, aldehydes, and carboxyl groups as they, when reacting with aldehydes or phenol, follow an identical reaction pathway with phenol and formaldehyde to form phenolic resin adhesives. By this, it can not only reduce the manufacturing costs but also contribute towards the reduction of harm formaldehyde-freer phenols free.

### 2.1. Lignin formaldehyde resin adhesive

Phenol-formaldehyde resin is one of the most commonly used adhesives for wood panel products, thanks to its remarkable strength, water resistance, and thermal stability. However, phenol is pricey since it is produced from fossil fuels. Additionally, the use of phenol-formaldehyde resins is restricted due to the toxicity of phenol and formaldehyde. Due to their comparable phenolic structures, researchers have tried substituting lignin for phenol in several cases. Lignin can be used in place of phenol to considerably lower production costs and lessen environmental impact. Because lignin contains functional groups such as phenolic and aliphatic hydroxyls, aldehydes, and carboxyl, it can undergo a reaction with both phenol and formaldehyde. Under alkaline conditions, the phenolic units of lignin may react with formaldehyde to generate methylol derivatives that condense internally or with other similar phenolics. Plenty of research has been conducted to prepare lignin-phenol-formaldehyde resins using different types of lignin (alkaline, kraft lignin, lignosulfonate, steam explosion, and pyrolysis lignin) as a substitute. Lignin was employed as a precursor to manufacture Fe_3_O_4_-lignin-based composites, which were used to synthesize an electromagnetic wave (EMW) absorbent adhesive suitable for plywood production. In line with the Chinese National Standard (GB/T 9846.32004), synthetic adhesives displayed appropriate viscosity, formaldehyde emission, and adhesive bonding strength.^37^ Briefly, the substitution of 20% of the lignin phenols by using Fe_3_O_4_ nanoparticles resulted in bonding strength of 0.73–0.76 MPa and formaldehyde emission of 0.22–0.36 mg L^−1^ for the bonded poplar plywood. Overall, Fe_3_O_4_@lignin (1 : 0.5)-phenol-formaldehyde adhesive displayed the highest EMV with a reflection loss of around −29.5 dB. In a similar study, lignin-phenol-formaldehyde (LPF) was synthesized using different types of lignin at varying concentrations ranging from 0 to 40 wt%. According to the results, all the examined lignin showed a rapid increase in LPF viscosity with increasing phenol substitution. Pine kraft lignin with 20% and 40% phenol substitution showed the highest LPF resole curing rate.^38^

The primary barrier to expanding the applications of lignin in phenolic adhesives is the low reactivity with formaldehyde than phenol. As a result, it has been shown that lignin must undergo certain modifications before it can be used in PF resin. Different lignin pretreatments have been demonstrated to partially overcome the low reactivity of lignin with formaldehyde. These strategies include ionic liquid treatment, glyoxalation, reduction, phenolation, *etc.* Younesi-Kordkheili and Pizzi^39^ studied the impact of lignin modification (glyoxal (G), phenol (P), ionic liquid (IL), and maleic anhydride (MA)) on the characteristics of LPF adhesives. According to the results, lignin treated with maleic anhydride exhibited good curing qualities, but LPF resins modified with maleic anhydride or ionic liquid showed fewer formaldehyde emissions and greater mechanical strength. Compared to unmodified lignin resin, the improvement in curing properties (cure at a lower temperature) of modified lignin resin can be ascribed to the high cross-link content accompanied by the acceleration effect. On the other hand, the flexural strength and modulus of maleated LPF-bonded panels were higher (2953 MPa) than those of unmodified LPF-bonded panels (2510 MPa and 17 MPa). These enhancements can be ascribed to the bond strengths and stiffness between maleated lignin (thanks to numerous reactive sites) and formaldehyde.

Several strategies have been proposed to increase the reactivity of lignin, which can be divided into three categories. These chemical modification approaches lead to the introduction of reactive groups on the lignin structure. However, these approaches frequently yield undesired byproducts. Physical approaches, such as microwave or hydrothermal treatments, use high temperatures and pressures to break the lignin structure, lower molecular weight, and expose new sites, enhancing the overall reactivity. However, the harsh reaction conditions limit its applicability at an industrial scale. The biological technique involves microorganisms and enzymes such as laccase to break down lignin; however, this approach is not viable due to longer reaction times and the cost and recovery of enzymes. Furthermore, such modification techniques necessitate the separation as well as the purification of modified lignin that entails multiple processing stages, raising the overall cost and limiting the large-scale application of LPF. Chen *et al.*^40^ used moderate conditions to demethylate lignin using NaOH/urea. Rather than being separated, all the resulting products were used together to manufacture demethylated LPF (DLPF) resin. The concentration of benzene ring's –OCH_3_ concentration has decreased from 0.32 mmol g^−1^ to 0.18 mmol g^−1^, while the functional group content of the phenolic hydroxyl group rose by 176.67%, enhancing the overall reactivity of demethylated lignin. The adhesion strength of the resulting DLPF resin was found to be according to acceptable standards. Furthermore, the particleboard panels joined with modified lignin LPF resins did not reveal a significant difference in terms of dimensional stability. The resultant LPF resins were characterized after lignin was modified using various techniques.

In lignin, the presence of multiple phenol groups with open *ortho* locations makes it a strong contender to replace fossil-based phenol. This can be ascribed to hydroxymethylation and polymerization by condensation with formaldehyde.^41^ Lignin has a long history of presence in PF resins.^42^ While it has structural and performance similarities with phenolic polymers, using lignin as an additive has certain limitations, such as dark color, heterogeneity, slow curing rate, and low reactivity. Furthermore, the use of lignin in resin compositions is sometimes hampered by poor addition rates and long curing durations.^43^ Due to the low solubility of lignin from common commercial sources in common solvents under non-alkaline or non-aqueous conditions, it becomes necessary to consider chemical modification or fractionation when producing phenolic resins. The goal is to produce consistent lignin fractions with defined qualities. To accomplish this, several fractionation procedures have been developed, assuring uniform lignin fractions with well-defined properties.^44^ Rodrigues *et al.*^45^ describe a method for fractionating kraft lignin (KL) to get low molar mass lignin fractions by using ethyl acetate. These fractions were utilized to partially replace non-renewable phenol in the manufacture of phenolic resins. After fractionation, the soluble lignin fraction (LFSol) showed better solubility and higher hydroxyl group concentration. Besides, LFSol-derived phenolic resins had similar adhesion and thermal stability to commercial resins. LFSol also outperformed other lignin fractions in replacing a considerable amount of commercial resin. According to the results, soluble LFSol displayed a higher rate of phenol substitution in the LPF resin than the insoluble component of KL in ethyl acetate.

Another common form of formaldehyde resins is urea-formaldehyde adhesives. In this, urea and formaldehyde are condensed to produce UF resin, which is extensively used in the manufacturing of composite materials and wood panels. Urea-formaldehyde resin accounts for 50% of the overall volume of adhesive manufacture, with an annual production of around 5 million tons. These adhesives have multiple limitations, such as formaldehyde emission and a low level of water resistance, despite their widespread use. To overcome these problems, researchers have used various modification techniques for UF resin, such as the incorporation of lignin. Lignin can be incorporated into UF resins as a practical way to make them eco-friendly and enhance their environmental performance.^46^ Additionally, the incorporation of lignin improves the thermal endurance, formaldehyde emissions, and waterproofing of wood panels.^47^ Similar outcomes were reported by Boussetta *et al.*,^48^ who added 10% bagasse cane lignin and 13% molasses beet lignin led to improved bonding strength and decreased formaldehyde emissions. However, in lignin-based resins, the increase in mechanical characteristics is very marginal because of lignin's poor reactivity. To overcome this, lignin modification and its copolymerization with UF resins have been the subject of substantial investigation. For instance, Kordkheili and Pizzi^49^ increased the mechanical performance of soda bagasse lignin by treating it with 1-ethyl-3-methylimidazolium acetate ionic liquid. Similar to this, Gonçalves *et al.*^50^ modified urea-formaldehyde resin with hydroxymethylated alkali lignin, achieving the best results for samples containing 30 wt% lignin. The modified resin had a low free formaldehyde level of 0.049% and a 4.56 MPa bonding strength.

### 2.2. Lignin formaldehyde-free adhesives

**Fig. 3:**
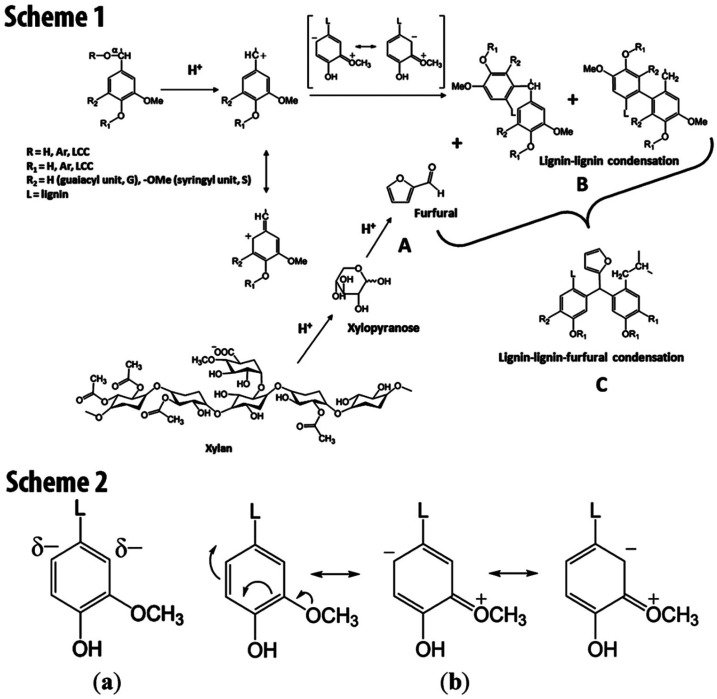
Proposed scheme of lignin-furfural condensation and resulting components; Scheme 1a: breakdown of xylose-based sugars, Scheme 1b: condensation of lignin–lignin, Scheme 1c: condensation mechanism of lignin–lignin-furfural; Scheme 2a: induction effect of the alkyl group at position 1, Scheme 2b: the resonance effect of the electron pairs on the methoxy oxygen, reproduced with permission from Klashorst, 1989, Copyright (1989) ACS.

A growing area of research focuses on the utilization of lignin to design green and sustainable formaldehyde-free adhesives. In this context, lignin-furfural has been recently reported as a lignin-formaldehyde-free adhesive. Furfural has the potential as a cross-linking agent and can produce condensation products with lignin (Scheme 1 in Fig. 3).^51^ It is proposed that lignin and furfural, which are often employed as substitutes for phenol and formaldehyde, respectively, in PF resins, can be utilized in the context of the lignin-furfural resin system. Under acidic conditions, a reaction occurs between lignin and furfural. An increase in electron density on the C_2_ and C_6_ carbons of the phenylpropanoid units under acidic circumstances is caused by two mechanisms, *i.e.*, the induction of the alkyl group at position-1 and the electron pairs’ resonance on the methoxy oxygen (Scheme 1 in Fig. 3).^52^ This leads to the condensation of the aromatic ring's C2 and C6 sites with the electrophilic aldehyde carbonyl carbon and turns into active sites for electrophilic substitutions.

A potential process for both lignin–lignin and lignin-furfural condensation is shown in Scheme 2 in Fig. 3. However, it should be emphasized that lignin may also be depolymerized (acidolyzed) in acidic conditions. The proton-induced elimination of water (ether) from the benzylic position results in the production of a carbonium ion at the location under acidic circumstances. The carbonium ion, therefore, undergoes further reactions as a result of the breakage of the –ether bond in the –O–4 structure, which produces Hibbert ketones in a manner reminiscent of an acidolysis process. Several studies have been carried out to prepare lignin-furfural adhesives. Dongre *et al.*^53^ used lignin extracted from sugar maple (Acer saccharum) in hot water to synthesize PF-free adhesive blends. The effect of pH levels (0.3, 0.65, and 1), *ex situ* furfural presence, and curing conditions on the tensile characteristics of glass fibers reinforced with the adhesive are investigated and compared to the reinforcing capabilities of commercially available PF resin. The adhesive blend created at a pH of 0.65 without the addition of furfural has the highest tensile characteristics and achieves 90% of the tensile strength of PF resin. According to Gosselink *et al.*,^54^ substituting a furan-based resin with unmodified grass-separated soda lignin up to 10% led to identical wood failure behavior. However, replacing the same quantity of periodate-activated lignin in the furan-based resin resulted in lower wood failure. This reduction can be ascribed to the existence of partly solubilized lignin particles in the acidic composition, which could impede the furan resin's crosslinking process. Furfural-lignin-based adhesives have been commercialized due to the low reactivity of lignin and furfural compared to formaldehyde and phenols.

Lignin has also been used to produce lignin-polyurethane adhesives. Polyurethanes can be produced by reacting polyols and poly or tri-isocyanates. Apart from the excellent adhesive strength and resilience, the poor biodegradability and high cost of production make polyurethane a less viable material. Incorporating lignin into polyurethane-based adhesive can contribute not only towards biodegradability but also reduce the overall cost of production as lignin is a cheap ingredient. Several studies have reported polyurethane-lignin-based adhesives for different applications. The physical properties of lignin-polyurethane adhesives can be easily tuned by changing different parameters, such as adjusting the molecular weight of lignin or using lignin-rich in aliphatic hydroxyl groups (modification of lignin through hydroxyalkylation and oxypropylation). The increased content of aliphatic hydroxyl groups is vital for the increased reactivity of isocyanate and lignin and, consequently, enhanced bonding strength.

Tannin has also been employed as a substitute for fossil phenol to synthesize lignin–tannin adhesive. Although the reactivity, structure, and properties of tannin depend upon the source of biomass, various tannins from different sources have displayed higher reactivity towards lignin, making them a desirable feedstock for producing wood adhesives (Table 1). High viscosity, low degree of cross-linking, and shorter life are among some of the disadvantages that come with tannin when used in lignin–tannin adhesives. Several studies have reported the use of tannin as a main component in lignin–tannin adhesives. Similarly, soy protein, an agro-biomass resource consisting of 20 different amino acids exhibiting different side chains, has been employed in lignin-based adhesives for wood. The side chains influence the hydrophilicity/hydrophobicity and reactivity of the adhesive with wood panels. Soy protein got the attention of researchers for adhesive production due to its renewability, abundance, and cost-effectiveness. Problems like high viscosity and hydrophilicity have been overcome by modifying soy protein through cross-linking, chemical denaturation, hydrolysis, *etc.*

**Table 1:** List of lignin-based adhesives for different applications, wood, biomedical, and packaging

Components	Lignin concentration	Application	Main outcomes	Ref.
Poly(butylene adipate-*co*-terephthalate) (PBAT), kraft lignin	Kraft lignin (0%, 1%, 3%, 5% and 10% by mass)	Food packaging	PBAT-kraft lignin (KL) films were produced as a laminated food packaging layer. KL enhances the hydrophobicity and polymer miscibility of the adhesive.	55
Ca^2^+-tannic acid@sulfonated lignin-polyacrylamide	0.25 gr sulfonated lignin with varying concentrations of tannic acid	Hydrogel for skin	Sulfonated lignin-polyacrylamide hydrogel revealed excellent conductivity, adhesion, and UV resistance.	56
Depolymerized kraft lignin and camelina protein		Protein-based green adhesive	H_2_O_2_-induced depolymerized kraft lignin was reacted with camelina protein to produce green adhesive. Water resistance and adhesiveness were improved.	57
Hydrogel matrix with demethylated catechol lignin and graphene oxide	2–4 wt%	Hydrogel for on-skin sensing	Increasing the concentration of lignin from 2 to 4 wt% led to enhanced adhesion and enhanced UV protection.	58
Kraft and organosolv lignin from Oil palm (1564 g mol^−1^)	Kraft lignin: activated free ring position 2.99% organosolv lignin: 2.06%	Green adhesive for wood	Kraft lignin with higher activated free ring sites has a higher potential for producing wood adhesive.	59
Depolymerized Kraft and organosolv lignins/bisphenol A(BPA)	25–100 wt% of depolymerized Kraft and organosolv lignin	Biobased epoxy resins	Depolymerized Kraft/Organosolv lignin blended with BPA to produce green epoxy resin. The thermal stability of epoxy resins has been significantly improved with increasing concentrations of lignin.	60
Lignosulfonic acid sodium salt (lignin)	0, 10, 25, and 50 wt%	Green adhesive for cork	Supplementing lignin into the adhesive formulation led to improved adhesion and reduced the bisphenol A concentration.	61
Kraft lignin from pulp and paper and glycerol diglycidyl ether from biodiesel	coarse, Fine, grounded, and coarse-grounded particles at 29%, 37%, 54%, and 54% respectively	Plywood formaldehyde-free adhesive	Resins were produced by using glyceride as a cross-linker with different types of lignin without any catalyst. The adhesion properties were recorded nearly like some of the formaldehyde resins.	62
Sodium lignosulfonate	0.1 gr sodium lignosulfonate	Lignin reinforced hydrogels	The presence of lignin enhanced the asymmetric adhesion, wettability, and conductivity of the hydrogels.	63
Hydroxypropylated lignin and castor oil (CO)	10 wt%, 20 wt%, and 30 wt%	Polyurethane (PU) synthesis	Hydroxypropylation of polypropylene carbonate with lignin using castor oil was explored. The resulting PU revealed improved adhesion and young modulus.	64
Lignin-based aromatic monophenols	0 wt%, 20 wt%, 50 wt%, and 80 wt% substitution	Lignin-based green epoxy adhesive	Monophenols *i.e.*, guaiacol, vanillin, and methyl guaiacol were used to substitute commercial BPA in the epoxy adhesive. Sample with 80 wt% lignophenols and 20 wt% BPA showed excellent adhesion properties.	65
Nano and microparticles of lignin in bulk formaldehyde resol	5 wt% and 10 wt%	Wood adhesive	Adhesive prepared by using micro and nano lignin particles favored thermal curing reaction and thermal resistance. The shear strength was enhanced from 8.7 to 10.9 MPa using 5 wt% lignin nanoparticles.	66
Lignin from *Solanum Elaeagnifolium Cavanilles*, urea, formaldehyde	Lignin to urea, formaldehyde ratio (*w : w*); 5 : 95, 10 : 90, 15 : 85 and 20 : 80	Wood adhesive	Adhesive containing 15 : 85 lignin displayed excellent mechanical properties and a significant decrease in the use of formaldehyde.	67
Lignosulfonate (LS), polyamidoamine-epichlorohydrin (FAI-Cl)	Lignosulfonate 100 mg mL^−1^	Underwater adhesive	Underwater implemented bio-adhesive was prepared by mixing LS and FAI-Cl. The obtained adhesive displayed instant adhesion in water curing even under high temperatures, alkali, and long soaking conditions.	68
Catechol from lignin and soybean protein	Lignin 0 to 4 wt%	Plywood adhesive	100% biobased adhesive produced from lignin and soybean protein resulted in a 101.4% improvement in the wet shear strength of the plywood. Besides, it also showed mildew and antibacterial resistance.	57
Lignin and furfural	15% furfural, 50% lignin	Wood adhesive	Furfural-modified lignin-phenol-formaldehyde revealed improved wet shear strength, low viscosity, better penetration to the wood surface, and stronger adhesion.	69
Lignin vitrimers	28 to 50 wt%	Aluminum and wood adhesives	Catalyst-free kraft lignin-poly (ethylene glycol) divinyl ether (PDV) adhesive was prepared using a one-pot synthesis approach (click). The resultant adhesive showed excellent lap shear strength and recyclability.	70
Lignin and chitosan	Lignin/chitosan ration; 1 : 2, 1 : 1, 2 : 1	Wood adhesives	Chitosan and lignin have been processed together to produce wood adhesive. The incorporation of chitosan has significantly affected the adhesion and water resistance.	71

In conclusion, the incorporation of lignin into adhesive formulations represents a pivotal step toward fostering sustainability across diverse industries. By replacing conventional petroleum-derived raw materials, such as phenol, formaldehyde, and polyether polyols, with lignin, industries can not only mitigate rising costs but also address environmental concerns associated with solid waste and gaseous pollutants during the production and use of fossil fuel-derived products. For instance, phenol-formaldehyde resins, widely used for their adhesion, hydrophobicity, and heat resistance, raise health and environmental concerns due to the toxic nature of phenol and formaldehyde. Lignin's structural similarity to phenol positions it as an ideal substitute, offering both economic and environmental advantages. However, challenges persist in optimizing lignin's reactivity with formaldehyde, prompting the exploration of various modification strategies. Chemical, physical, and biological approaches aim to enhance lignin's performance, with each method presenting its own set of advantages and limitations. Chemical modifications, such as glyoxalation and phenolation, offer increased reactivity but may yield undesired byproducts. Physical treatments, including microwave or hydrothermal methods, enhance overall reactivity but face limitations in industrial scalability. Biological techniques involving enzymes, though environmentally friendly, suffer from longer reaction times and cost considerations.

The quest for lignin's widespread application in adhesives extends beyond phenol-based resins to urea-formaldehyde adhesives. Researchers explore lignin's potential to improve the environmental performance of urea-formaldehyde resins, addressing issues such as formaldehyde emissions and water resistance. Modification techniques, such as ionic liquid treatment, highlight efforts to enhance lignin's mechanical properties within urea-formaldehyde formulations. An emerging area of interest involves lignin's role in the development of formaldehyde-free adhesives. Lignin-furfural systems, under acidic conditions, present an eco-friendly alternative with potential cross-linking capabilities. Moreover, lignin's integration into polyurethane and tannin-based adhesives holds promise for creating biodegradable alternatives, addressing concerns related to poor biodegradability and high production costs associated with traditional polyurethane adhesives.

In light of the progress made, key challenges persist, including the need for standardized processes, scalability of modification techniques, and addressing performance limitations such as curing rates and color issues. Collaborative efforts among researchers, industries, and regulatory bodies are imperative for overcoming these challenges and establishing robust safety standards. The future of lignin-based adhesives depends on continuous advancements, expanding application possibilities, and a collective commitment to sustainable practices. As industries strive for a low-carbon economy, lignin stands as a beacon of promise, offering a renewable and eco-friendly alternative that can revolutionize the adhesive landscape.

## 3. Lignin contribution to thermal properties of polymer composites

Lignin and lignin-based materials have demonstrated the ability to enhance the thermal properties of composites through various mechanisms. The remarkable thermal stability and UV resistance of lignin can be attributed to its intrinsic aromatic structure, making it a key factor in these properties. Furthermore, lignin and lignin nanoparticles serve as thermal stabilizers by effectively absorbing and dissipating heat. The reduced size of the nanoparticles enables them to infiltrate the polymer matrix, resulting in improved dispersion and interfacial bonding, thereby enhancing thermal conductivity and stability. Additionally, lignin nanoparticles act as nucleation sites for crystallization, further augmenting the thermal properties of the composites.^72,73^

### 3.1. Lignin contribution to thermal behavior of polyethylene composites

Lignin and lignin particles can influence the thermal properties of polyethylene/lignin composites through several mechanisms. One of the key mechanisms is the interaction between lignin/lignin nanoparticles and the polyethylene (PE) matrix. Lignin can form hydrogen bonds with polyethylene matrix, which can reduce the mobility of polyethylene chains, leading to an increase in the melting temperature (*T*_m_) and the glass transition temperature (*T*_g_) of the composite. Additionally, lignin can act as a nucleating agent during the crystallization process of polyethylene, promoting the formation of smaller and more uniform crystals, which can increase the degree of crystallinity of the composite and further enhance its thermal stability. Furthermore, incorporating lignin and lignin particles into composites facilitates the formation of a char layer during the combustion process, which impedes the rate of thermal degradation and enhances the fire resistance.^74^ Overall, the incorporation of lignin and lignin particles into a PE matrix can improve the thermal properties of the composite by promoting better interfacial adhesion, enhancing the crystallization behavior of polyethylene, and increasing the fire resistance of the composite through the formation of a char layer.

There are several research works performed on improving the thermal stability of PE. For instance, Sadeghifar *et al.*^75^ conducted a study to investigate the effects of methylation and lignin fractionation on the thermal stability and antioxidant properties of polyethylene-lignin blends. The authors found that the lower molecular weight fraction of kraft lignin exhibited higher antioxidant activity compared to its acetone-insoluble counterpart. However, the complete methylation of phenolic hydroxyl groups in lignin led to no antioxidant activity, highlighting the significance of these groups in imparting antioxidant properties. The researchers also examined the impact of lignin and its fractions on the thermal stability of PE and its mixes. The addition of unmodified kraft lignin and its lower molecular weight fraction to polyethylene increased the oxidation induction temperature (OIT_temp_) values by approximately 50 °C relative to pure polyethylene. However, further increments in lignin content did not provide additional enhancements in OIT_temp_ values. The OIT_temp_ of pure medium-density PE was found to be approximately 206 °C. The introduction of unfractionated kraft lignin resulted in a noteworthy increase in OIT_temp_ values. The highest observed OIT_temp_ value reached around 260 °C, representing an increase of nearly 50 °C compared to neat PE when 5% of original unfractionated lignin was incorporated. However, the utilization of the acetone-insoluble fraction of kraft lignin to formulate a 5% PE blend exhibited a comparatively lower stabilizing effect on the OIT_temp_ of the blend, with an increase of only 23 °C. The study also noted that the incorporation of methylated lignin into polyethylene blends resulted in increased OIT_temp_ values, likely due to the formation of an aromatic char from lignin that hinders polyethylene degradation and raises the thermal degradation temperature of polyethylene.

### 3.2. Lignin contribution to thermal behavior of rubber-based composites

Lignin can significantly impact the thermal properties of natural rubber (NR) blends or composites. This is due to the strong intermolecular interactions between the lignin or lignin nanoparticles and the natural rubber matrix, which enhance the crosslinking density and restrict the mobility of the rubber chains. The incorporation of lignin nanoparticles can also improve the dispersion of the filler in the rubber matrix, leading to a more homogeneous structure and better mechanical properties. However, the optimal amount and type of lignin or lignin nanoparticles to be added to natural rubber blends or composites depend on the specific application and desired properties. The surface chemistry and particle size of lignin nanoparticles can also affect their compatibility with the natural rubber matrix and the resulting thermal and mechanical properties of the composites.

In a study by Hosseinmardi *et al.*,^76^ lignin was utilized to produce natural rubber-lignin nanocomposites. Organosolv lignin (OSL) particles are dispersed in de-ionized water and dissolved by adjusting the pH to 10.5 using an aqueous ammonia solution. After 20 minutes of mixing, a homogeneous dispersion is achieved. Meanwhile, natural rubber latex is stirred, and its solid content is adjusted to 45 wt% with an aqueous ammonia solution at pH 10.5 (for control) or an alkali aqueous OSL dispersion (for nanocomposite films). Stirring continues for 1 hour at 35 rpm, followed by 24 hours at 12 rpm for a homogeneous dispersion and de-aeration. The mixtures are cast into flat glass molds, left at room temperature overnight to form dry films (300–400 μm thickness), and further cured at 110 °C for 6 hours under a gentle flow of dry nitrogen. To simulate industrial latex dipping conditions, the samples are dusted with calcium carbonate powder to prevent tackiness and leached with hot de-ionized water (65 °C for 10 min) before curing. This assesses the effects of common industrial steps on the final film characteristics. The dynamic and morphological analysis confirmed the homogeneous dispersion of nano-lignin in natural rubber. The incorporation of lignin nanoparticles improved the dispersion of lignin in the natural rubber matrix and resulted in a significant improvement in the mechanical and thermal stability, as well as the glass transition temperature of the resulting nanocomposite. In contrast to micro-sized lignin, the incorporation of nano-sized lignin demonstrated improved thermal stability of the resulting nanocomposite while maintaining mechanical properties.

**Fig. 4:**
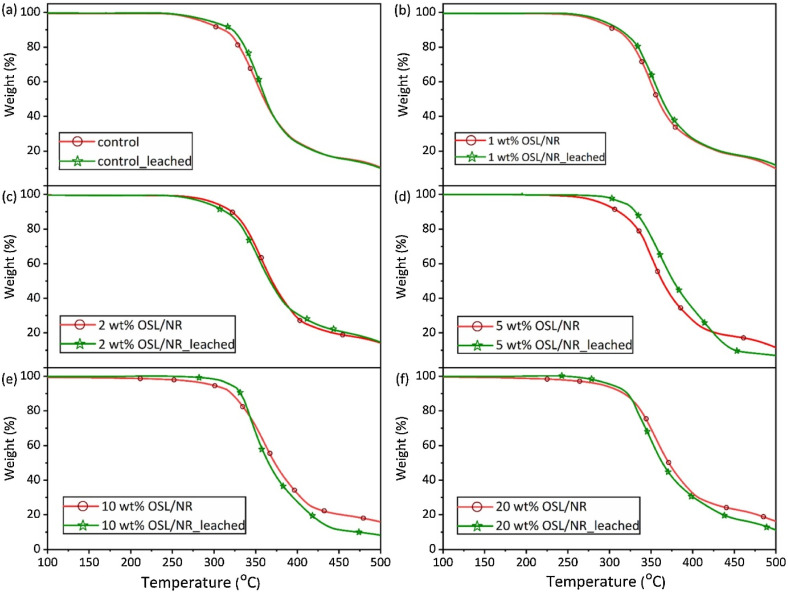
The Thermogravimetric analysis (TGA) curves of (a) control, (b) 1 wt% OSL/NR, (c) 2 wt% OSL/NR, (d) 5 wt% OSL/NR, (e) 10 wt% OSL/NR and (f) 20 wt% OSL/NR samples, with and without dusting and leaching processes.^76^ (a–f) were reproduced with permission from Hosseinmardi, 2021, Copyright (2021) Elsevier.

Fig. 4 illustrates the thermal characteristics of pure natural rubber OSL/lignin nanocomposites under the dusting and leaching processes. The acquired data indicate that the thermal performance of both control and OSL/NR nanocomposites, reinforced with OSL up to 2 wt%, experienced no significant impact from the processes of dusting and leaching (Fig. 4a–c). These findings validate the effective dispersion, distribution, and crosslinking of OSL within the NR matrix, demonstrating its stability after leaching. However, as depicted in Fig. 4d–f, the thermal degradation behavior of OSL/NR nanocomposites with 5 wt%, 10 wt%, and 20 wt% OSL was notably influenced by the leaching process. The *T*_onset_ exhibited an increase in leached nanocomposite samples with 5 wt% and 10 wt% OSL, indicating initial thermal decomposition occurring at a higher temperature. This slight elevation could be attributed to the potential removal of other low molecular extractives and improved interparticle integration. Additionally, the residual mass at 500 °C for dusted and leached samples with higher OSL loading (5–20 wt%) decreased compared to their counterparts, likely due to the removal of lignin from these samples.^76^ They have also shown that both the control and OSL/NR nanocomposites were thermally stable up to 240 °C and the onset decomposition temperature (*T*_onset_) increased with increasing OSL content (up to 254 °C for 20 wt% OSL/NR).

### 3.3. Lignin contribution to thermal behavior of poly (vinyl alcohol) composites

Lignin and lignin nanoparticles can also have an impact on the thermal properties of Poly (vinyl alcohol) (PVA) in their blends or composites. When lignin or lignin nanoparticles are added to PVA, they can form hydrogen bonds with the PVA molecules, leading to increased intermolecular interactions and stronger interfacial adhesion between the lignin and PVA phases. The mechanism behind this effect is related to the chemical structure of lignin, which contains various functional groups such as hydroxyl, carboxyl, and methoxy groups that can interact with PVA molecules *via* hydrogen bonding. The hydrogen bonds formed between the lignin and PVA can restrict the motion of the PVA chains, leading to an increase in the thermal stability of the resulting blends or composites. Furthermore, the incorporation of lignin nanoparticles into PVA can also result in increased surface area and greater dispersion of the filler in the matrix, leading to a more homogeneous structure and improved thermal properties. However, the optimal amount and type of lignin or lignin nanoparticles to be added to PVA depend on the specific application and desired properties. The particle size, surface chemistry, and morphology of lignin nanoparticles can also affect their compatibility with PVA and the resulting thermal and mechanical properties of the composites. Therefore, further research is needed to optimize the processing conditions and composition of lignin-based PVA composites.^77^

The study conducted by He and colleagues in 2019^78^ employed citric acid-modified lignin nanoparticles (LNPs) as a reinforcing filler in PVA. They used a solvent casting approach to add etherified and esterified lignin nanoparticles and unmodified LNP to PVA at loading levels up to 10%. The modified LNPs exhibited excellent dispersion in the PVA matrix without any visible phase separation. The modified LNPs and PVA formed intermolecular hydrogen bonds as a result of the etherification and esterification processes. The enhanced antioxidant properties of the modified LNPs are likely due to their specific characteristics, such as a high level of phenolic compounds, narrow polydispersity, and low molecular weight. The nanocomposites displayed higher decomposition temperature and maximum degradation, indicating improved thermal stability. The PVA-MLNP system exhibited a slight increase in thermal stability compared to PVA-LNP due to the improved dispersion of modified LNPs in PVA and reduced agglomeration of modified LNPs. PVA exhibited a first maximum thermal degradation temperature (*T*_m1_) of 251.2 °C, contrasting with the temperatures of 279.7 °C and 280.3 °C observed for PVA composites containing LNP and MLNP at a concentration of 10 wt%, respectively. The predominant loss, accounting for approximately 69%, transpired in neat PVA within the temperature range of 210 °C to 310 °C, a phenomenon attributed to the decomposition of the polymer. The nanofillers also enhanced the UV protection of PVA, with citric acid-modified LNPs being more effective than unmodified LNPs. The mechanical and thermal characteristics of the resultant composite were improved because the alteration of LNPs increased their interfacial contact with the PVA matrix. The increased hydrogen bonding interactions between the modified LNPs and PVA contributed to these improvements.

**Fig. 5:**
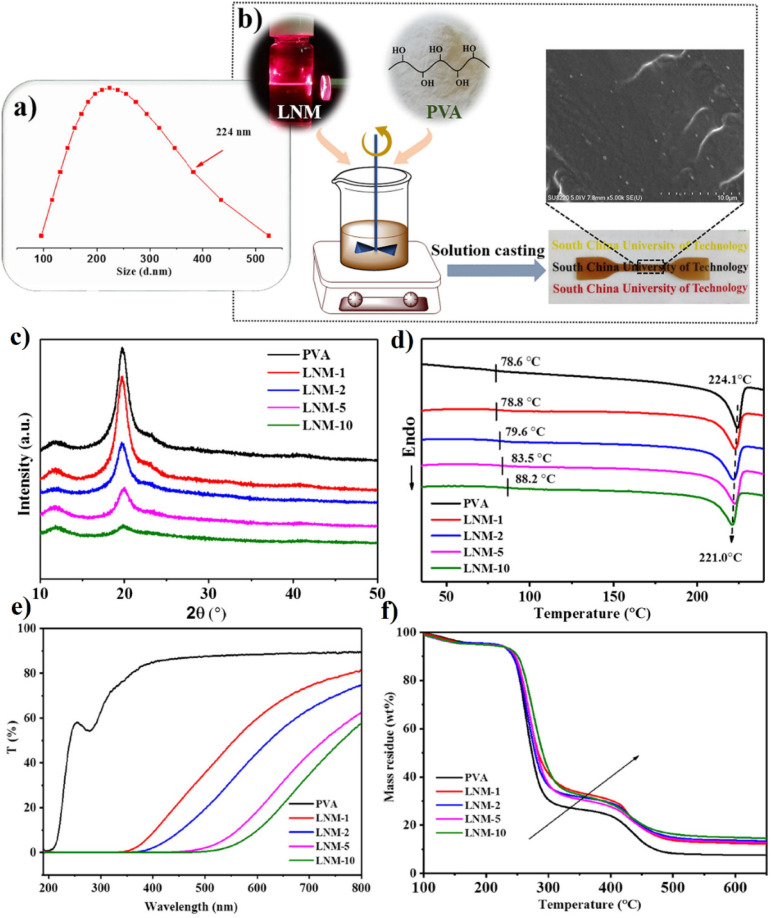
(a) LNM particle size distribution. (b) Schematic representation of the PVA/LNM composite film preparation technique. (c) XRD spectra of the nanocomposites. (d) DSC melting curves of the prepared samples. (e) UV–vis transmittance curves of the nanocomposite films. (f) TGA curves of PVA and PVA/LNM nanocomposites at N_2_ atmosphere.^79^ (a–f) Were reproduced with permission from Zhang, 2020, Copyright (2020) Elsevier.

Zhang *et al.*^79^ conducted a study where they synthesized lignin nanomicelles (LNMs) with an average size of 44 nm using a solvent evaporation method, as depicted in Fig. 5. These LNMs were then incorporated into PVA to fabricate PVA/LNM composites with improved UV shielding and thermal stability properties. The addition of LNM to PVA resulted in an increase in the maximum thermal decomposition rate temperature (*T*_max_) from 262.3 °C to 273.5 °C, indicating improved thermal stability. This is due to the PVA matrix's and LNM's strong intermolecular hydrogen bonding interactions, which limited the flexibility of PVA chain segments. The presence of aromatic structural units in LNM molecules was also found to contribute to the enhancement of thermal stability. Due to PVA's melting temperature being so near to its decomposition temperature, melt processing makes it challenging to produce products with complex forms. The incorporation of LNM significantly improved the thermal properties of PVA nanocomposite films, providing a promising approach to producing PVA composite materials *via* melt processing. This study highlights the potential of lignin-based nanomaterials for improving the properties of polymer composites. When the LNM content reached 10 wt%, Fig. 5c and d illustrates a noticeable decrease in the crystallinity of LNM-10. This observation was substantiated by DSC analysis. While the melting temperature (*T*_m_) of the LNM/PVA nanocomposite films remained nearly constant (224.1 °C for pure PVA, 221.0 °C for LNM-10), the crystallinity obtained from DSC analysis decreased from 29.2% for pure PVA to 22.8% for LNM-10.

### 3.4. Lignin contribution to thermal behavior of poly (methyl methacrylate) composites

Lignin and LNPs can act as thermal stabilizers by providing additional crosslinking and hydrogen bonding to the polymethyl methacrylate (PMMA) matrix, which can enhance the thermal stability of the nanocomposite. This can result in a higher decomposition temperature and a reduced rate of weight loss upon heating. Additionally, lignin can act as a reinforcement filler, increasing the mechanical properties of the PMMA nanocomposites. Lignin and LNPs can improve the thermal conductivity of the PMMA nanocomposites. This can enhance the heat dissipation from the nanocomposite, leading to improved thermal stability. They can reduce the free volume in the PMMA matrix, which is the space between polymer chains, by forming hydrogen bonds with the PMMA matrix. A reduction in free volume can lead to increased density and improved thermal stability.^80^

**Fig. 6:**
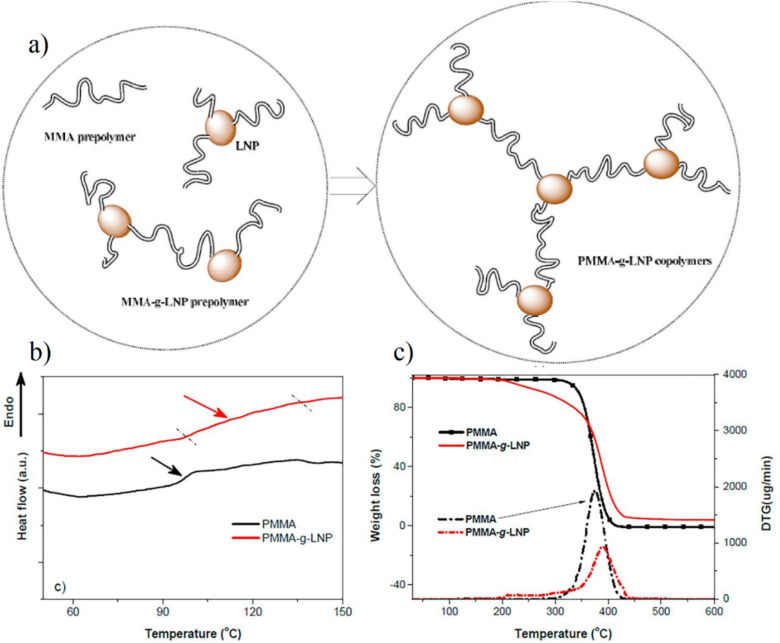
(a) Schematic image of the polymerization process. (b) DSC scan of PMMA and PMMA-g-LNP. (c) TGA and DTG curves of PMMA and PMMA-g-LNP.^21^ (a–c) Were reproduced with permission from Yang, 2018, Copyright (2018) Elsevier.

Yang *et al.*^21^ conducted a study in which lignin nanoparticles (LNPs) were incorporated into PMMA to create nanocomposites known as PMMA-g-LNP. The thermal characteristic of PMMA-g-LNP nanocomposites was analyzed through TGA and DSC, as depicted in Fig. 6. The glass transition temperature of the PMMA-g-LNP system (110 °C) was found to be greater than that of commercial PMMA (100 °C), which might be attributed to the two polymers’ different molecular weights. Moreover, the transition of PMMA-g-LNP was broader than commercial PMMA. TGA was performed to investigate the thermal stability of the two polymers under a nitrogen atmosphere. The differential thermogravimetry (DTG) profile of PMMA-g-LNP was less sharp than commercial PMMA, suggesting a more gradual thermal degradation. Furthermore, the *T*_max_ of PMMA-g-LNP rose from 373 °C to 386 °C, demonstrating that LNP enhanced PMMA's thermal stability. It is worth noting that a distinct peak was found for the DTG curve of PMMA-g-LNP polymer from 200 °C, which was connected to the breakdown of LNP. The thermal decomposition of LNP gradually initiated within this temperature range, resulting in a lower *T*_onset_ compared to commercial PMMA.

In conclusion, the incorporation of lignin and lignin-based materials into polymer composites has shown promise in enhancing thermal properties across various matrices. The ability of lignin to form hydrogen bonds, act as a nucleating agent, and improve interfacial adhesion has led to improvements in thermal stability, mechanical properties, and fire resistance. However, challenges such as optimizing composition, understanding long-term stability, and addressing environmental concerns remain. The future of lignin-based polymer composites holds great potential, with continued research and development expected to overcome current limitations and unlock new opportunities for sustainable and high-performance materials.

While the progress in incorporating lignin into polymer composites for thermal enhancement is evident, several challenges and areas for future exploration persist. Optimization of the amount and type of lignin or lignin nanoparticles for specific applications remains a critical aspect. The influence of lignin characteristics such as particle size, surface chemistry, and morphology on compatibility with different polymer matrices needs further investigation. Additionally, the scalability and cost-effectiveness of lignin-based composites should be addressed for practical applications.

## 4. Lignin contribution to barrier properties of polymer composites

**Fig. 7:**
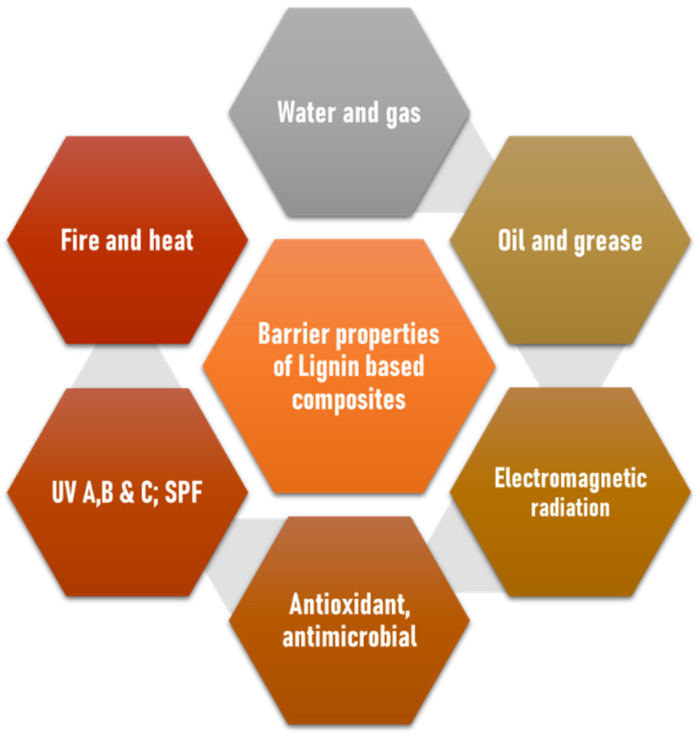
The barrier properties of the lignin-based composites are discussed in this section.

In recent times of industrialization, films, and coatings have played a very important role in protecting a wide variety of perishable and nonperishable products for long-term storage and transportation. To become good packaging materials, these films and coatings must be thermally and mechanically robust and possess excellent barrier properties. Barrier properties can be imparted by lignin in composites,^81^ as discussed in this section, Fig. 7. Conventional petrochemical-derived synthetic polymers such as polypropylene, polyethylene, polyethylene terephthalate, polyamide, and so on exhibit excellent barrier properties that give rise to exceptional resistance towards moisture/water, gaseous (mainly oxygen), chemical and biological entities that made them a popular choice in industrial applications. This robustness and stability, in turn, make them nonbiodegradable, and they end up in landfills after a single use and also pose a threat of leaching harmful chemicals like Bisphenol A, micro/nanoplastics, *etc.*, into nature.^82^ That makes it essential to find or create sustainable bio-based alternatives with equal or better efficiency and affordability for their usage in commercial applications. Research reports that lignin appears to be a prospective candidate for single or multicomponent coatings owing to its abundance, chemical functionalities, ease of modification, and decent to excellent barrier properties. A polymer can show high or low barrier properties depending on its chemical structure (functional groups, polarity, chain length, branching, *etc.*), reactivity, and morphological aspects like crystallinity, voids, and so on. For example, the antioxidant and antimicrobial properties of lignin are due to its side chain structure and high polyphenolic hydroxyl content that is a result of the kraft pulping process and is responsible for the scavenging action of their phenolic structures on oxygen-containing reactive-free radicals. Lignin contains plenty of conjugated aromatic rings, carbonyl groups, C=C, quinines, and methoxy-phenoxy groups, and these unsaturated functional groups accelerate the light-catalyzed photoreactions leading to the formation of quinones and chromophoric bodies that activate the UV blocking property of lignin.^83^ The antimicrobial action of pristine lignin has been interpreted to occur due to the presence of a double bond in α and β positions in the side chain and –CH_2_ group in γ position of phenolic contents that can act as ionophores, thus increasing the ion permeability across the cell membrane causing cell death, lignin nanoparticles are known for penetrating the bacterial cell membranes, and produce of ROS resulting in the depletion of ATP and decrease in intracellular pH of the cells.^84^ Lignin has also been found to be an interesting bio-based alternative (component) to halogenated flame retardants owing to its aromatic structure and thermal behavior. While in inert conditions, thermal degradation of lignin leads to the formation of stable and protective charred carbon layer residues over any substrate (also called intumescence) that acts as a condensed phase, forming a barrier to oxygen and diffusion of other combustible gases.^85^ This char may thermally degrade in the presence of oxygen, so its thermal stability must be reinforced by its chemical modification or the composite formation to enhance its intumescent character due to its inflammability.^86^

**Fig. 8:**
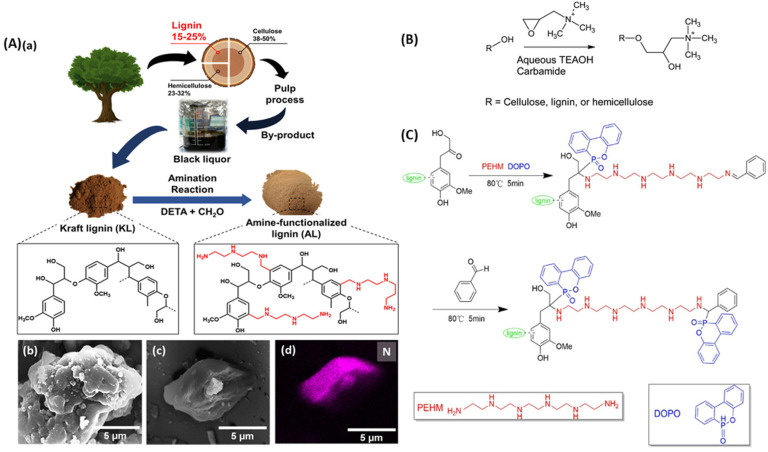
(A) Scheme showing the production of AL by aminating lignin. FE-SEM images of (b) KL and (c) AL particles with (d) corresponding EDS elemental mapping showing the distribution of N, reproduced with permission from Chung, 2023, Copyright (2023) ACS; (B) reaction scheme of the cationization of sawdust, reproduced with permission from Sirviö, 2020, Copyright (2020) ACS; (C) synthesis of modified lignin (NP-L) with pentaethylene hexamine (PEHM) and 9, 10-dihydro-9-oxa-10-phosphaphenanth-rene-10-oxide (DOPO), reproduced with permission from Zhou, 2022, Copyright (2022) Taylor & Francis.

Chung *et al.*^87^ developed amine-functionalized lignin (AL), as shown in Fig. 8A, to be incorporated in rubber to improve its antiaging properties, thermal stability, and ozone/fatigue resistance through the radical scavenging (antioxidant) effect. They studied and compared the effect of AL with that of other antioxidants such as 6PPD (*N*-(1,3-dimethylbutyl)-*N*′-phenyl-*p*-phenylenediamine) and kraft lignin on the characteristics of the modified rubber product and found that the former imparts superior resistance to thermal and ozone aging than others. The AL particles promote the vulcanization reaction of rubber by activating sulfur, which increases the curing rate and cross-link density of rubber, making it more fatigue-resistant.

Sirviö *et al.*^88^ cationized sawdust lignocellulose using four different solvents containing tetraethylammonium hydroxide with different carbamides (urea, methylurea, ethylurea, or dimethylurea) with glycidyltrimethylammonium chloride as the cationization agent, shown in Fig. 8B. The product was mechanically disintegrated to obtain uniform suspension for making transparent films (about 80% at 600 nm), high UV absorption (transmittance <1%), and OTR (below 400 and 4000 m^3^ μm m^−2^ day atm at R.H of 50 and 92%, respectively). The chemical structure of lignin contains a large number of active sites such as –OH, –OCH_3_, or phenolic hydroxyl groups, which can be easily functionalized by phosphorus and/or nitrogen-containing groups, several metal ions, and silica groups for obtaining enhanced flame retardancy (FR) properties. Modified lignin serves distinct functions based on the type of modification. N-modified lignin operates through a gas phase flame-retardant (FR) effect by releasing water vapor and NH_3_. These substances effectively absorb surface heat and dilute flammable fumes emitted during thermal decomposition. On the other hand, P-modified lignin primarily acts in the condensed phase by generating potent acids. This modification promotes dehydration and carbonization on the substrate's surface, further enhancing its fire resistance. P/N-modified lignin shows a synergistic effect by improving the thermal stability of the substrate, while metal ions and silica incorporation help with their high specific heat capacities and incombustible nature, thereby promoting the formation of a thicker char layer. Zhou *et al.*^89^ modified enzymatically hydrolyzed lignin by chemically grafting phosphorus (2.95%) and nitrogen (3.55%), as shown in Fig. 8C using phenolic condensation reaction and the product showed excellent flame retardancy and smoke suppression performance, where 5% replacement of phenolic foam by modified lignin brought down the mean heat release rate (MHRR) from 20.83 kW m^−2^ to 12.33 kW m^−2^.

Hult *et al.*^90^ esterified lignin with salts of palmitic and lauric acids and used it as a coating on the paperboard. A significant decrease in water vapor transmission rate (WVTR) and oxygen transmission rate (OTR) of 40 g m^−2^ and 1750 cm^3^ m^−2^, respectively, were observed for paperboard coated with 10.4 g m^−2^ hardwood kraft lignin palmitate when measured for 24 h. Lignin ester-coated paperboard showed substantially lower WVTR due to the hydrophobic character of the lignin derivatives, with palmitate coating working better than laurate, to be specific, as compared to fatty acids-coated boards.

**Fig. 9:**
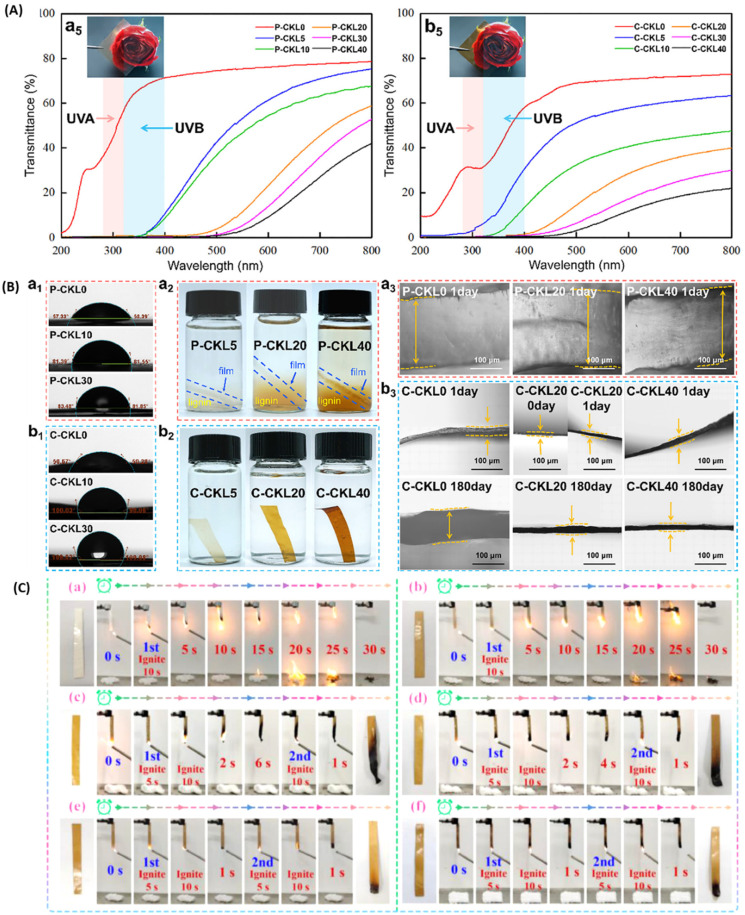
(A) Transmittance curves of the composite films; (B) Water contact angle of the physical (a1) and chemical (b1) composite films. Photographs of the physical (a2) and chemical films (b2) soaked in water for 24 h. Cross-section of the physical (a3) and chemical films (b3) soaked at different times, reproduced with permission from Ma, 2022, Copyright (2022) Elsevier (C) UL-94 test photos of (a) polyacrylonitrile (PAN), (b) L/C/PAN, PAN samples treated with (c) 5, (d) 8, (e) 10, and (f) 12 wt% Phytic Acid solution, reproduced with permission from Guo, 2022, Copyright (2022) ACS.

Xing *et al.*^91^ developed transparent biodegradable films with a blend of polymers with lignin having improved UV barrier properties. Soda lignin derivatives were obtained by grafting 10-undecenoic and oleic acids using solvent- and catalyst-free processes. These derivatives and neat lignin were then melt-blended with PBAT to prepare films for complete absorption in the UV range (280–400 nm) up to 50 h and possess good thermal stability. The undecanoyl lignin variant showed higher UV absorption and better compatibility with the PBAT matrix but had lower thermal stability than the oleoyl-treated counterpart. Another example of a multicomponent composite film is a polylactic acid/lignin (PLA/LG) blend separately modified with two graft copolymers (PLA-g-GMA and PLA-g-PEGMA). The effect of two methacrylate variants and lignin content on the gas barrier, interfacial adhesion, and thermal and mechanical properties of PLA/LG composites were observed. PLA-g-PEGMA grafted PLA/LG showed an 86% reduction in O_2_ permeability compared to the neat PLA film and showed a superior gas barrier to PLA-g-GMA grafted composite due to its higher crystallization capability.^92^ The cellulose/glucomannan/lignin blend that was chemically crosslinked using epichlorohydrin resulted in mechanically robust composite films that could withstand a tensile stress of 110.47 MPa. The effect of lignocellulose composition was investigated on desired properties and was also compared with that of films produced by physical mixing of the components (no chemical crosslinking). 20 wt% lignin loaded composite could block >99% of UV A & B, showed a water contact angle of 110.38°, and a greater water barrier with no swelling after being soaked for 180 days, as shown in Fig. 9(A & B). Chemical crosslinking was found to be an important influence on the firm and uniform internal structure of the films that enhanced the UV barrier property and prevented self-aggregation that was observed in the physical films.^93^

Rodriguez-Melendez *et al.*^94^ synthesized lignin-based ‘green’ and intumescent flame retardant by making a blend of lignin, casein, and polyacrylonitrile (PAN) followed by linking the blend with different wt.% of phytic acid. The multicomponent FR samples were tested by limiting oxygen index (LOI), UL-94, and cone calorimetry. The total period of burning and the length of char residue indicated that 10 wt% PA solution imparted a good charring effect that acts as a physical barrier and can further delay the flame spread and retard the burning speed. This was witnessed in the UL-94 test, where FR-PAN-10 formed a swelling char in the ignition area and quickly self-extinguished after the flame was removed, as shown in Fig. 9C. Cone calorimetry proved that the phytic acid-treated polymer mixture not only reduced HRR effectively but also suppressed smoke production when compared to the untreated control polymer blend samples.

**Fig. 10:**
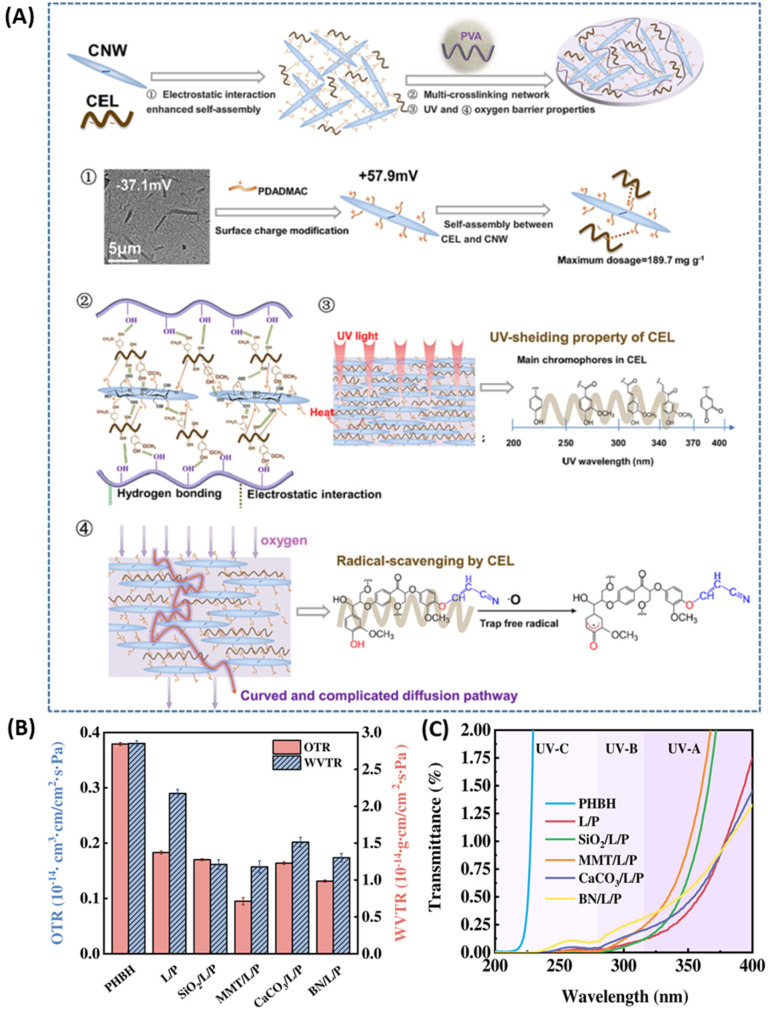
(A) Schematic diagram showing the procedure of composite film production and its structural details leading to UV and oxygen shielding, reproduced with permission from Tian, 2023, Copyright (2023) Elsevier. (B) OTR, WVTR performance and (C) UV-visible spectra of PHBH, lignin/PHBH (L/P), and INPs incorporated composites, reproduced with permission from Zeng, 2022, Copyright (2022) Wiley.

Tian *et al.*^95^ developed a liquid mulching composite film inspired by a layered structure of mussels with water-soluble reagents such as cyanoethylated lignin/modified cellulose nanowhiskers/PVA through self-assembly. The mussel-inspired structure significantly enhanced its overall barrier performance towards antioxidant, oxygen, and UV barrier properties, shown in Fig. 10A. Cellulose nanowhiskers modified with poly (diallyl dimethyl-ammonium chloride) (PDADMAC) were bonded to cyanoethyl lignin, which was further blended with PVA to form a suspension film, which prevented degradation of pesticides and improved water resistance of soil, thereby promoting seed germination and seedling growth. Inorganic nanoparticles of silica, montmorillonite, calcium carbonate and boron nitride were used as fillers in the blend of lignin and poly-3-hydroxybutyrate-3-hydroxyhexanoate (PHBH) to observe their effect on the barrier and other properties of the resulting composites by Zeng *et al.*^96^ Four nanocomposite thin films of thickness 30 to 50 μm were obtained and tested for oxygen and water vapor permeability and UV resistance. The oxygen gas barrier performance of lignin/PHBH composites was improved by 7%, 48%, 11%, and 28%, and their water vapor barrier performance was improved by 44%, 46%, 30%, and 40% upon adding the fillers SiO_2_, MMT, CaCO_3_, and BN nanoparticles, respectively (refer Fig. 10B). This not only improved the mechanical and thermal stability of the nanocomposites but also exhibited less than 1% transmittance in UVA, UVB, and UVC regions, as shown in Fig. 10C. The incorporation of nanoparticles increased the crystallinity of the material, and tighter crystal structure lowered the intermolecular gaps, resulting in lower permeability and higher barrier properties of the nanocomposites.

Composite made of polylactic acid embedded with silver nanoparticles where organosolv lignin served as reducing agent organic solvent resulted in homogeneous films, was developed by Shankar *et al.*^97^ PLA/lignin/AgNPs composite films were tested for UV-light barrier, water vapor permeability, and antibacterial, mechanical and thermal properties. The UV barrier of neat PLA was observed by measuring transmittance at 280 nm, giving a value of 67.7 ± 8.5%, which decreased to 0.5 ± 0.1% after the incorporation of lignin and AgNPs. The WVP value of the neat PLA film was 2.93 ± 0.21 × 10^–11^ g m m^−2^ Pa^−1^ s^−1^, which reduced to 2.45 ± 0.21 × 10^–11^ g m m^−2^ Pa^−1^ s^−1^ for the composite film due to a strong intermolecular interaction between PLA biopolymer chains and more hydrophobic organosolv lignin to form a tortuous path for the water vapor diffusion through the polymer matrix.

There are quite a few reports on PLA/cellulose nano fibers (CNF)/lignin bio-nanocomposites explored for improved barrier properties. Nair *et al.*^98^ used nanofibrillated cellulose with high lignin content (NCFHL) extracted from the red cedar (Thuja plicata) bark to mix with PLA latex to get Petri dish-casted airdried films followed by heating and compression molding. The addition of 10 wt% of the NCFHL increased the modulus and strength by 88% and 111%, respectively, and the water vapor transmission rate was reduced by 52% compared to neat PLA. Wang *et al.*^99^ also prepared biodegradable PLA/CNF/lignin bio-nanocomposites but with pulps having different lignin contents *i.e.*, low (DP-L), medium (DP-M) and high (DP-H) lignin and studied the effect of the composition on the barrier properties. Water Vapor Transmission of PLA/LCNF-H composite film reduced to one-third of that of pure PLA film, while LCNF-H with 19.5% lignin content into PLA resulted in full blocking of the UVB and UVC and most of the UVA blocking for the composite. Song *et al.*^100^ produced a fire-resistant paper combining graphene nanoplatelets (GnP), carbon nanotubes (CNTs), and lignin in different proportions and coated using a Meyer rod to test its gas permeability and fire resistance. Air resistance was studied by Gurley number (s/100 ml) where the plain paper had 154 ± 3s, lignin-coated paper 202 ± 12s, and nanocomposite-coated paper went up to 1717 ± 240 s as the pores of the cellulose matrix got blocked with carbon nanomaterials and a further reduction in permeability observed with increasing the coating thickness. GnP/CNT mixture synergistically reduced the extent of burning and weight loss than those coated by each component separately. However, going from high to low content of GnPs showed improvement in flame-retardancy with up to 14.6% and 15.7% reductions in the burned area and weight loss, respectively. Also, the nanomaterial-coated paper exhibited self-extinguishing properties, exclusive of the lignin content, while only lignin-coated paper did not show such behavior.

**Fig. 11:**
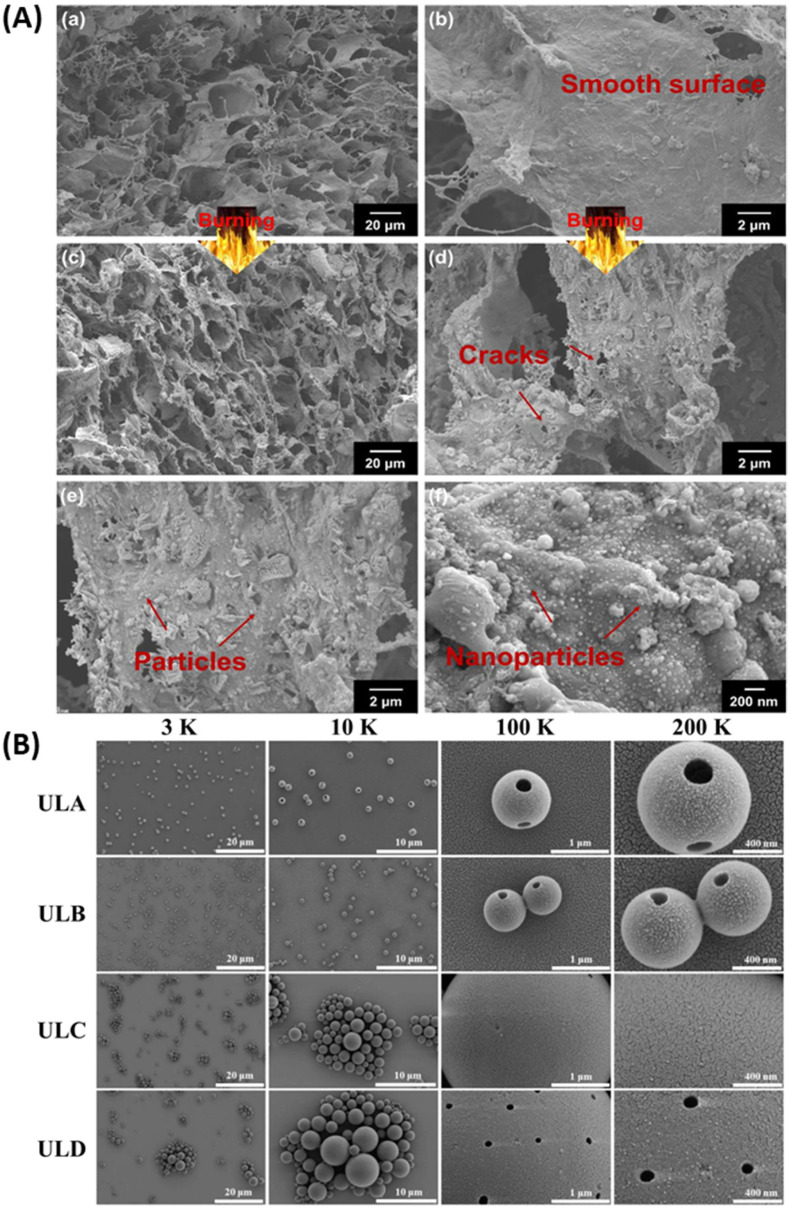
(A) Morphology of PGM-3 aerogel before and after flame tests. PGM-3 aerogel showing (a) original microstructure and (b) smooth surface before, and (c and d) rough surface after flame tests. (f) The nanoparticles surfaced on the aerogel after flame tests, reproduced with permission from Li, 2022, Copyright (2022) Elsevier. (B) SEM images of LNPs obtained from various lignin fractions, reproduced with permission from Zhang, 2023, Copyright (2023) Elsevier.

Another example of biodegradable UV shielding film made ofcore–shell lignin–melanin nanoparticle (LMNP) compounded with poly(butylene adipate-*co*-terephthalate) (PBAT) was also studied for its photostability by Xing *et al.*^101^ Neat PBAT shows optical transmittance of 66% at 550 nm, has poor UV shielding in the UVA range (320–400 nm), but absorbs 92% of UVB light (280–320 nm) and below due to its benzene rings and carbonyl groups. While the transparency of the PBAT nanocomposites drastically reduces, UV absorption increases to more than 98% of UVB and 93–98% of UVA with 5 wt% of nanoparticle loading. As far as photostability is concerned, samples containing 0.5–2.0 wt% LNP kept their tensile strength (σ) and elongation at break (ε) even after 40 h of UV exposure, but samples with 5 wt% LNP could not retain its mechanical properties. Li *et al.*^102^ developed another lignin-based ternary nanocomposite by mixing phosphate-treated nanofibrillated lignocellulose, gelatin, and MXene to result in highly porous and ultralight aerogel (PGM), showing fire retardant and EMI shielding properties. To be specific, PGM-3 aerogel blocked electromagnetic waves above 99%. Plenty of mobile charge carriers in d-Ti3C2Tx nanoflakes, large electric dipoles, and their conducting structural interfaces contributed to the EMI shielding by PGM hybrid aerogels at large. Also, incident electromagnetic waves suffered multi-reflections in the porous architecture, enhancing wave absorption. As far as the flame-retardant performance is concerned, the composite aerogel retained its size and morphology compared to phosphate lignocellulose nanofibrils during initial combustion. The former showed an LOI of 32% and PHRR of 30.1 W g^−1^, while the latter gave PHRR (62.7 W g^−1^) and LOI (28%), which can be considered as hard-combustible materials according to ISO 4589–2 (LOI > 27%). Using its various components, PGM-3 could form both solid as well as gas phase barriers towards obstructing heat, flame, and flammable gases, thereby protecting the aerogel from fire, as shown in Fig. 11A. Moreover, MXene nanoflakes can detect a rapid change in the electrical resistance due to heat, which can act as a trigger for fire-warning signals.

In another example, Zhang *et al.*^103^ produced bioactive packaging films made of lignin nanoparticles derived from deep eutectic solvents used as nanofillers in the chitosan matrix. The composite film showed improved tensile mechanical characteristics (an increase of up to 40%) and reduced swelling and water transmittance properties owing to the incorporation of LNPs. 10 wt% LNP content not only dramatically enhanced the UV shielding but also the free radical scavenging antioxidant ability from 20 (only chitosan) to 90%. Antioxidant and antibacterial properties of related packaging materials can prolong the shelf-life of foodstuff.^104^ Lignin shows antibacterial activity. Moreover, the refrigerated (4 ± 1 °C) grass fish wrapped with composite films revealed better quality, and the shelf life was extended to 10 days. In another study, the utility of lignin nanoparticles in sun protection was tested where the lignin nanoparticles were produced using fractionated ultrafiltration lignin of four kinds of molecular weights, as shown in Fig. 11B. The LNPs showed narrow size distribution (0.8–1.4 m), high dispersibility (PDI = 1.41), good antioxidant properties (89.47%, 5 mg mL^−1^), high brightness (ISO% = 7.55), and high lightness indices L* value (L* = 72.3). Upon loading 5% ULNP to sunscreen, its sun protection factor (SPF) value increased from 14.93 to 63.74.^105^

In conclusion, the incorporation of lignin in polymer composites presents a promising avenue for enhancing the barrier properties of films and coatings, making them suitable for various industrial applications. Lignin, derived from renewable sources, offers a sustainable alternative to conventional petrochemical-derived polymers. The unique chemical structure of lignin, including conjugated aromatic rings, carbonyl groups, and phenolic hydroxyl groups, contributes to its diverse functionalities, such as antioxidant, antimicrobial, and flame retardant properties.

Advantages of lignin-based composites include their potential for creating environmentally friendly, biodegradable materials with improved barrier properties. Lignin's ability to form stable and protective charred carbon layers under inert conditions enhances flame retardancy. Additionally, modifications like cationization, esterification, and grafting enable tailoring lignin properties for specific applications, such as improving antiaging, thermal stability, and flame retardancy in rubber. Researchers have explored various formulations of lignin-based composites, including blends with polymers like poly(butylene adipate-*co*-terephthalate) and polylactic acid, as well as nanocomposites with nanoparticles like silver, cellulose nanofibers, and graphene. These formulations exhibit enhanced UV resistance, water vapor barrier properties, and mechanical strength, making them suitable for applications such as packaging, mulching, and fire-resistant coatings.

However, challenges and issues persist. The variability in lignin's properties based on its source and extraction method necessitates careful consideration in composite design. Furthermore, the economic viability and scalability of lignin-based composites for large-scale industrial use require attention. Addressing these challenges will be crucial for realizing the full potential of lignin in sustainable packaging and coating materials. Looking ahead, future research should focus on optimizing lignin extraction methods, exploring innovative modification techniques, and scaling up production processes to make lignin-based composites more competitive in terms of cost and performance. Additionally, continued efforts in developing multifunctional lignin-based composites with tailored properties for specific applications will contribute to the advancement of sustainable materials in the industrial sector. Overall, the integration of lignin into polymer composites holds promise for creating environmentally friendly materials with enhanced barrier properties, contributing to a more sustainable future.

## 5. Lignin contribution to antimicrobial properties of polymer composites

Lignin contributes to the antimicrobial properties of composites by incorporating its inherent antimicrobial compounds. When lignin is incorporated into composites, it imparts antimicrobial functionality, which can inhibit the growth and proliferation of bacteria, fungi, and other microorganisms on the composite surface.^106,107^ The mechanism activated by lignin to create this antimicrobial function in composites is multifaceted. Lignin's antimicrobial activity arises from the presence of various antimicrobial compounds, such as phenolic compounds and quinones, within its chemical structure. These compounds possess inherent antimicrobial properties and can disrupt the cellular structures and metabolic processes of microorganisms. When incorporated into composites, lignin acts as a natural antimicrobial agent. The antimicrobial compounds present in lignin are released or exposed on the composite surface, creating a hostile environment for microorganisms. Lignin can inhibit the growth and colonization of bacteria, fungi, and other microbes, helping to mitigate microbial contamination and degradation of the composite material. The antimicrobial compounds present in lignin can penetrate the cell walls of microorganisms, interfere with their membrane integrity, inhibit enzymatic activity, and disrupt essential metabolic processes. These actions ultimately lead to the inhibition of microbial growth and the prevention of biofilm formation on the composite surface. The antimicrobial properties of lignin-based composites can find applications in various industries where microbial contamination is a concern, such as healthcare, food packaging, and agricultural applications. By incorporating lignin into composites, these materials can offer a natural and sustainable solution for preventing microbial growth and preserving the integrity and functionality of the composite in environments prone to microbial contamination. Ongoing research aims to optimize the incorporation and distribution of lignin within composites to maximize its antimicrobial effectiveness and expand its potential applications.^108,109^

To the best of our understanding, chitosan is recognized as one of the most important polymers with antimicrobial properties. It is frequently combined with lignin to create novel antimicrobial substances. In a study conducted by Kim *et al.*,^110^ they developed nanoparticles made of lignosulfonate and chitosan by mixing their respective solutions in the presence of an organic phase derived from vegetal oil. The formation of these nanoparticles was driven by strong electrostatic interactions between the two oppositely charged polymers. This resulted in the creation of core–shell particles with a highly positively charged surface. Interestingly, the lignosulfonate-chitosan nanoparticles demonstrated improved antimicrobial activity against both Gram-negative and Gram-positive bacteria, surpassing the effectiveness of pure lignosulfonate or chitosan nanoparticles, while maintaining their cytotoxicity at the same level.

**Fig. 12:**
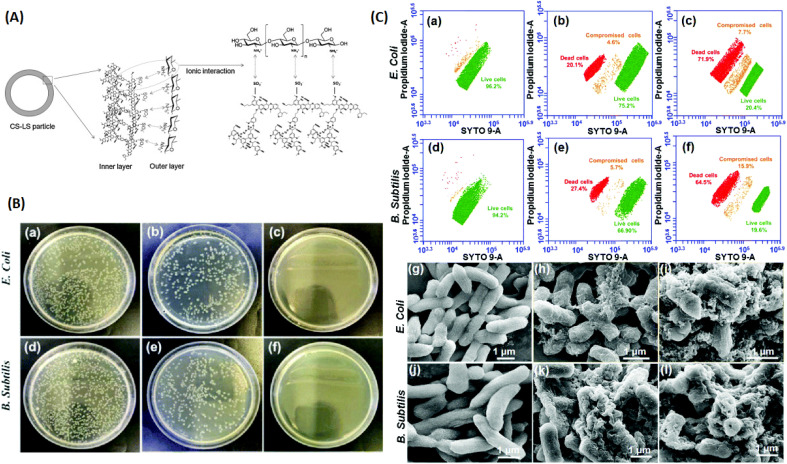
(A) Scheme of chitosan–lignosulfonate particles formation due to ionic interactions, reproduced with permission from Kim, 2013, Copyright (2013) Elsevier. (B) Antimicrobial assay of the nanoparticle by susceptibility test. (C) Antimicrobial assay of the nanoparticle by flow cytometry and SEM images of antimicrobial nanocomposites. Reproduced with permission from Pandey, 2019, Copyright (2019) Royal Society of Chemistry.

Pandey *et al.*^111^ conducted studies where they produced a chemically cross-linked composite of lignosulfonate and chitosan through a Mannich reaction, as depicted in Fig. 12. This cross-linked composite exhibited superior antimicrobial efficacy in comparison to the ionic complex formed by lignosulfonate and chitosan. The heightened antimicrobial properties of the composite were ascribed to the close arrangement of the two polymers through covalent bonding, leading to an augmented positive charge on the surface. This, in turn, resulted in significant disruption of the bacterial cell membranes, leading to increased release of lactate dehydrogenase.

Due to the outstanding film-forming capabilities of chitosan and the zwitterionic nature brought on by the presence of amino and sulfonate groups, the combination of lignosulfonate and chitosan also has the power to generate a thin layer with antifouling qualities. As a result, this composite may be used to protect surfaces by successfully limiting bacterial adhesion and the production of biofilms, which lowers bio-corrosion. Notably, the research found that using the lignosulfonate chitosan composite as a coating over carbon steel reduced the sulfur content, a key bio-corrosion product, by 31%. Additionally, an electrochemical impedance study showed that lignosulfonate chitosan composites demonstrated a remarkable 84% prevention of carbon steel corrosion under dynamic flow circumstances.^112^

In conclusion, the incorporation of lignin into polymer composites presents a promising avenue for conferring antimicrobial properties to materials, offering a natural and sustainable solution to microbial contamination challenges in industries such as healthcare, food packaging, and agriculture. The multifaceted antimicrobial mechanism activated by lignin, involving the release of compounds disrupting microbial structures and metabolic processes, showcases its potential to inhibit microbial growth and prevent biofilm formation on composite surfaces. Ongoing research focuses on optimizing the incorporation and distribution of lignin within composites to maximize antimicrobial effectiveness and broaden potential applications. Furthermore, the combination of lignosulfonate and chitosan enhances antimicrobial properties, leading to the development of versatile composites with improved efficacy against bacteria and antifouling qualities. Future progress lies in advancing the understanding of lignin's antimicrobial mechanisms, optimizing formulations, and exploring novel applications, with a crucial emphasis on scalable production methods and cost-effectiveness for widespread industrial adoption. As research continues, lignin-based antimicrobial composites stand poised to revolutionize industries reliant on effective microbial control, providing sustainable solutions to address pressing contamination concerns.

## 6. Lignin contribution to the rheological properties of polymer composites

**Fig. 13:**
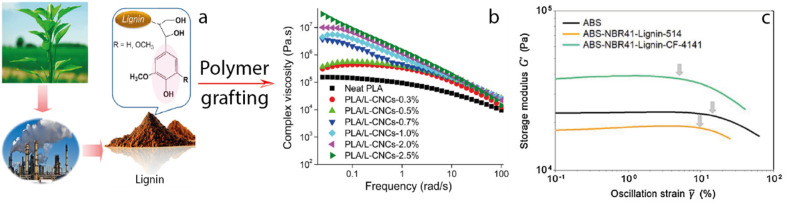
(a) Lignin structure, (b) Complex viscosity as a function of frequency, and (c) storage modulus as a function of strain. (a) Reproduced with permission from Zong, 2017,^125^ Copyright (2018) American Chemical Society. (b) Reproduced with permission from Gupta, 2017, Copyright (2017) American Chemical Society. (c) Reproduced with permission from Nguyen, 2018, Copyright (2018) Elsevier.

The elastic solid that complies with Hooke's law is treated by the conventional theory of elasticity. In deformations, the stress is inversely proportional to the strain and is independent of the rate of strain. Contrarily, hydrodynamics considers viscous liquids in accordance with Newton's law. The stress is independent of the strain and directly related to the rate of strain. However, most materials have viscoelastic qualities, which combine elasticity and viscosity. Rheology establishes a link between deformation, stress, and time; it also establishes a link between molecular structure and viscoelastic characteristics and material structure.^113^ Lignin (Fig. 13a) added as filler alters the physiochemical properties as well as reduces the price of the end product.^114^ Lignin is included in various applications, including a substitute for phenolic resins, cross-linker in epoxies incorporation with polyolefins, and so on.^115^ Due to its advantageous qualities, including optimal mechanical properties, chemical stability, ease of production, and recyclable nature, acrylonitrile butadiene styrene (ABS) is an extensively used engineering thermoplastic. ABS resin may have good interfacial compatibility with lignin since ABS polymer chains feature strong polar-CN groups and aromatic structures in the styrene and acrylonitrile segments, respectively. The use of lignin as a filler in ABS resin would increase its efficiency while increasing the use of biomass, making ABS partially biodegradable. More importantly, the presence of lignin increased the melt viscosity of ABS, resulting in increasing the polymer relaxation time, therefore, a better mixture of lignin and ABS and, as a result, higher physicomechanical properties.^116^

The most observed phenomena due to the presence of lignin are an increase in storage and loss moduli as well as an increase in the relaxation time. Chauhan *et al.*^117^ reported that molecules could pass one other more freely if the lignin in the liquid is rotated lengthwise in the flow direction. Due to the chemical cross-linking in their networks and the filler effect of the lignin, lignin-isocyanate polymer blends (10 and 15 wt% lignin content) were shown to have larger storage and loss moduli compared to isocyanate itself. Chauhan *et al.*^117^ and Gupta *et al.*^118^ reported the shear thinning effect of lignin and reported on PLA/lignin-coated cellulose nanocrystal composites (Fig. 13b). They suggested that lignin coating not only increased the compatibility of the PLA/cellulose nano crystals (CNC) mixture but also prevented the reaggregation of crystals in the molten polymer. Both studies stated that the shear thinning behavior resulted from the disentanglement and orientation of polymeric chains in the flow direction, therefore reducing the viscous behavior. Nguyen *et al.*^119,120^ also reported in (Fig. 13c) that the presence of lignin alone and lignin/carbon fiber in the ABS matrix resulted in a low strain amplitude, at around 10% and 5%, respectively, to retain the elastic characteristic. They expected that the two lignin-based composites’ heterogeneous architectures and oligomeric nature would cause each aggregated phase to exhibit a different local relaxation and dynamic response state. Moreover, the inflexible carbon fiber and poor flexibility of the lignin structure both considerably contributed to the strain-dependent plateau *G*′. Also, due to the presence of lignin and its covalent cross-linking with the polymer, the specimen showed a low phase angle and solid-like behavior. Dörrstein *et al.*,^121^ however, presented another interesting fact regarding the presence of lignin in the composites. They were expected to observe significant changes in viscosity as a result of increasing the lignin content in the composites; however, only 32% increase was exhibited. This can be explained by the fact that the local shear rate is greater than the total shear rate immediately around a particle. The rise in viscosity is somewhat impacted by this effect. Furthermore, while viscosity increased significantly from 24 000 Pa s to 47 000 Pa s by increasing the lignin content from 50 wt% to 62.5 wt%, a measured value viscosity of 12 600 Pa s related to the highest lignin loading (75 wt%) was observed and constituted the lowest value of all the examined samples. These findings are connected to a modification in the morphology of the lignin phase at heavily loaded composites. Lignin likely displayed self-plasticization at very high-volume fractions to create the matrix. In another story, Feng *et al.*^122^ observed that when the frequency was more than 10 rad s^−1^, the storage modulus exhibited frequency-independent behavior, indicating that the printed composite underwent a solid-like viscoelastic transition. The addition of a lignin/CNF mixture resulted in a decrease in storage modulus due to the interference with the polymerization of methyl acrylate resin during stereolithography. The aggregation of lignin/CNF in the printed composites, which hampered the transition of molecule chains and produced a high storage modulus, is likely to blame for the storage modulus’ increase with increasing lignin/CNF content from 0.1% to 0.5%. There was also a sign of shear thinning behavior in the printed composites. With the addition of lignin/CNF, the shear thinning behavior diminished, indicating a weak interaction between lignin/CNF and methyl acrylate matrix. High lignin/CNF content, however, would make up for this loss by aggregating in the matrix, which, as was already indicated, prevented the transition of methyl acrylate chains. Kim *et al.*^123^ also observed similar results. For instance, the comparison between the various PLA-based composites showed that the introduction of lignin to the matrix resulted in the decrease of storage modulus; however, the loss modulus did not show meaningful differences. This was evidenced as the loss modulus was believed to be less sensitive to the internal composition of materials compared to the storage modulus. Interestingly, the addition of lignin to the composites causes the shear viscosity to drop when compared to the pure PLA sample and the other composites. Song *et al.*^116^ reported that by increasing the lignin loading and compatibilizer incorporation, both storage and loss modulus rise in the low-frequency region. They proposed that this is caused by the dispersed phase particles’ deformability, which is typically seen for many multiphase systems even if they are still stiffer than acrylonitrile butadiene styrene. With increased lignin loading and compatibilizer addition, the two relaxation times of ABS blends demonstrate a similar tendency, which is to grow. Nevertheless, increasing lignin loading results in substantially longer relaxation times. Mixing 5% lignin just causes a slightly longer relaxation time of 0.33 s compared to 0.28 s for ABS. Moreover, as the amount of lignin loading increases, the zero-shear viscosity likewise rises. Practically speaking, these modifications entail that lignin limits polymer chain mobility while also shifting the frequency of droplet deformation in the terminal zone to a lower value. Cao *et al.*^124^ also showed that by increasing the lignin/polyhedral oligomeric silsesquioxane (POSS) content within the PLA-based matrices, the complex viscosity deteriorated significantly, indicating the lubricant-like effect of lignin/POSS. The storage modulus showed independence from the lignin mixture; therefore, the lignin has almost no effect on relaxation behavior. All matrices also showed similar results for loss modulus except the pure PLA. Regardless, the lignin/POSS lubricant increased the processability of the PLA matrix.

Liu *et al.*^126^ reported that the storage modulus of composites gradually decreased with increasing lignin dose, which can be attributed to the solid-like behavior brought on by the presence of hardwood flour. The storage modulus marginally increased as lignin content increased, indicating that the resultant composites had greater shear thinning behavior as a result of lignin softening. Lignin may act as a hybrid filler between the polyethylene matrix and the wood flour, facilitating the dispersion of the wood flour within the polymer matrix. Moreover, the wood and polymer may be joined together by the melt lignin, which is significantly more friendly with wood than polyolefin by entangling polyethylene and penetrating wood flour gaps. Generally, it is expected to have non-Newtonian pseudoplastic fluid characteristics from such composites, and the increase in the lignin content resulted in a decrease in the complex viscosity. The presence of lignin facilitated the melt flow within the extrusion and prevented the aggregation of wood flour inside the matrix. Due to the presence of lignin, the solid-like characteristic was diminished, and the composite displayed improved flexibility and, therefore, eventually showed higher mechanical properties. Lee *et al.*^127^ looked at the synergistic effect of lignin on composites. It is reported that lignin resulted in a decrease in shear viscosity while increasing the lignin content introduced the shear thinning effect in the system. Within their system, it is reported that the lignin/polypropylene played the compatibilizer role inside the PLA matrix; therefore, they showed significant storage moduli compared with those without lignin/polypropylene.

The effect of the lignin on the rheology of composites is very much dependent on various factors, including the polymer matrix, whether there are other fillers in the system or not, and so on. Also, it can be evidenced from the precious efforts that by reaching the correct threshold of lignin content within the composite matrix, the overall characteristics of the composites can be significantly improved. In Table 2, the general effects of lignin on the rheology of composites are exhibited.

**Table 2:** The effect of lignin on the rheology of different composites

Composite			Effect on Viscosity				Ref.
Polymer	Lignin (source)	Other fillers (compatibilizer)	Storage modulus (G′)	Loss modulus (G′′)	Complex viscosity	Overall effect	
High-density Polyethylene (HDPE)	Straw – enzymatic hydrolysis	Wood flour – maleic anhydride grafted HDPE	In the absence of wood flour increased/in the presence of wood flour decreased	Not specified	In the absence of wood flour increased/in the presence of wood flour decreased	Limiting the movement of Polymer chain/facilitating the dispersion wood floor in polymer matrix	126
PLA	Organosolv	Epoxycyclohexyl isobutyl polyhedral oligomeric silsesquioxane (POSS)	Independent of lignin-POSS/no effect on relaxation time	Lower than PLA itself/lignin-POSS caused better processability	Significant decrease by increasing the Lignin-POSS content	Lignin-POSS showed lubricant effect/reducing the entanglement of PLA chain	124
PLA	Not specified	MCC	Decrease	No meaningful effect	Decrease	Better processability	123
Methacrylate (MA)	Lignin-containing cellulose nanofibrils (L-CNF)	—	First addition decreased then increased by increasing the content	Not specified	Decrease	Decreasing Shear Thinning/weak interaction between L-CNF and MA	122
Acrylonitrile butadiene rubber	Hardwood-Organosolv	Un-sized PAN based carbon fibers (CF)	Neat lignin decreased the modulus/Lignin-CF increased	Not specified	Not specified	Lignin brought more solid-like behavior/Low damping	119
Ethylene-vinyl acetate (EVA)	Grass (poacae) silage-Organosolv	—	Not specified	Not specified	Increased continuously with increasing lignin	Lignin has a plasticization effect on composite	121
PLA	Spray-dried lignin-coated cellulose nanocrystals (L-CNCs)	—	The addition of L-CNCs led to a significant increase	The addition of L-CNCs led to a significant increase	First increasing gradually then dramatically	Disappearance of terminal flow/restricting the motion of PLA chains	118
4,4′-Diphenyl methane diisocyanate-MDI	Soda	PEG	Increased	Increased	Increased	Filler effect/increasing the stress stability	117
PLA-PP	lignosulphonate	coffee powders	Higher than the rest	Higher than the rest	The addition of lignin decreased the viscosity	High lignin resulted in shear thinning/lignin acts as a compatibilizer	127
PBAT	Corncob	TPS	First decreasing then increase	First decreasing then increase	Plasticization increased	Compatibilizer and lubricant role – improving the melt flow – plasticization effect	128
PP	lignin sulfonate	Carboxylated nitrile rubber emulsion + Acryl amide	Increased	Increased	Decreasing by increasing the lignin content	Formation of a heterogeneous network structure in the melt/increased relaxation time	129
PLA	Betula alba dark	MWCNT	First decrease then increase	Gradual increase	Not specified	Plasticizer effect/Improved the 3D printing	130
HDPE	Alkali (nanoscale lignin reverse micelles)	—	Decreased by increasing the lignin	Decreased by increasing the lignin	Decreased by increasing the lignin	Reducing the chain entanglement of HDPE/improving the processability of HDP	131
Natural rubber	Kraft, organosolv and soda	Polybutadiene rubber	Not specified	Not specified	Decreased by increasing the lignin	Plasticization effect/improving the efficiency of the processing of rubber compounds	132
PLA	Corn stover-derived Organosolv	—	Not specified	Not specified	Decreased by increasing the lignin content	Caused unfirm blending and shear thinning – better processability – Plasticization effect	114
ABS	Wheat straw alkali	SEBS-g-MA	Increased by increasing the lignin content	Increased by increasing the lignin content	Increased by increasing the lignin content	Increased the relaxation time/retarding the relaxation of polymer chains	116
Polystyrene	Softwood Kraft	Triblock copolymer, based on styrene and ethylene/butylene (SEBS)	Increased by increasing the lignin content	Increased by increasing the lignin content	Increased by increasing the lignin content	limiting the mobility of the polymer chains/Blend elasticity decreased	133
HDPE	Lignin removal from the cell wall of wood particle	—	Decreased drastically	Decreased drastically	Decreased drastically	Delignification resulted in non-terminal, solid-like rheological behavior	134
PHB	Pinus radiata wood chips-mechanical and enzymatic	—	Not specified	Not specified	Lower than neat PHB but no other differences	Higher lignin facilitated the 3D printing/better layer adhesion	135
poly(hydroxybutyrate) (PHB)	Bagasse-Soda	—	First decrease then increase	First decrease then increase	Decreased	Increasing the processability	136
Epoxidized natural rubber (ENR)	Industrial-sulfate	Zinc oxide, stearic acid, N-*tert*-butyl-2-benzothiazole sulfonamide, 2, 2′-dibenzothiazoledisulfde and sulfur	Not specified	Not specified	Increased	Ring-opening reaction on rubber	137
Polyamide (PA)	Lignin-cellulose fiber (LCF)		Increased by increasing the LCF content	Not specified	Increased by increasing the LCF content	Restricting the deformation of polymer/decrease in mobility of the polymer	138
ABS	Softwood-Kraft	Poly (ethylene oxide)	Not specified	Not specified	Decreased	Not specified	139
Poly(butylene adipate-*co*-terephthalate) (PBAT)	Sodium lignosulfonate nanoparticle	Maleic anhydride	Decreased	Decreased	Decreased	Decreasing the entanglement of polymer chains/decreasing the shear-thinning effect/plasticizer role	140
PLA	Ethyl acetate-treated lignin nanospheres	—	Lower than the Pure PLA	Lower than the Pure PLA	Decreased significantly	Decreasing the entanglement of polymer chains/significantly improving 3D printing	141

In conclusion, the integration of lignin into polymer composites has significant implications for their rheological properties. The conventional theories of elasticity and hydrodynamics, governing elastic solids and viscous liquids, respectively, are insufficient to describe the viscoelastic nature of most materials. Rheology emerges as a crucial tool, linking deformation, stress, and time, and establishing connections between molecular and material structures. The addition of lignin as a filler not only modifies physiochemical properties but also reduces the cost of the final product. Its versatility finds applications in various domains, such as a substitute for phenolic resins and a cross-linker in epoxies with polyolefins. Studies indicate that lignin influences the rheological properties of composites by facilitating the flow of molecules, preventing reaggregation of crystals, and introducing shear-thinning behavior. The synergistic effects of lignin in different systems, such as lignin/polypropylene acting as a compatibilizer, further highlight its versatile role. However, the effects are insignificant and depend on factors like lignin dosage and its interaction with other components in the composite.

Looking ahead, advancements in understanding the intricate interplay between lignin and polymer matrices are crucial. Optimizing the threshold of lignin content within composites can lead to significant improvements in their overall characteristics. Challenges lie in achieving a balance that enhances flexibility, mechanical properties, and processability while avoiding undesirable effects. The future perspective involves continued research to unravel the complexities of lignin's impact on rheology, paving the way for enhanced, sustainable polymer composites across diverse applications. Collaborative efforts in this direction will contribute to the development of eco-friendly materials and processes in the ever-evolving landscape of polymer science and technology.

## 7. Lignin contribution to mechanical properties of polymer composites

Hybridization has been used to enhance the properties of thermoplastic and thermoset polymers. Various additives have been utilized in hybrid materials, such as carbon-based particles, clay, metal oxides, silicon dioxide, and bio-based fillers. In the case of bio-based fillers, not only can the properties of the polymers be improved, but also their sustainability, green content, and carbon footprint.^142,143^ Lignin, in particular, has gained significant attention in recent decades due to its unique chemical structure that can be modified through various methods, its availability, its strong effect as reinforcement material, biodegradability, and renewability.^144^

**Fig. 14:**
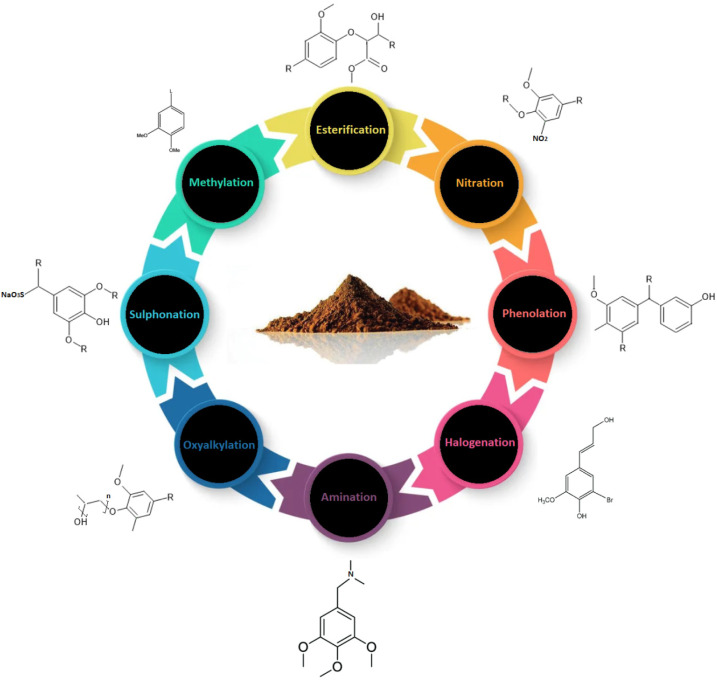
The most common lignin modifications.

The compatibility between lignin and most polymers is a most important subject, given the typical hydrophobic nature of polymers in contrast to the relatively less hydrophobic character of lignin extracted from plants, attributed to its abundant hydroxyl groups. Therefore, several chemical and physical approaches have been proposed to modify lignin to enhance its dispersibility and interface adhesion in composites. These modification methods often involve substituting the hydroxyl groups of lignin with hydrophobic moieties, Fig. 14. The chemically modified lignins not only find applications in composites but also meet the demands of various other fields, including coatings, *etc*.

In this section, we review reports focusing on the mechanical properties of thermoplastic and thermoset polymers reinforced with lignin particles. Among thermoplastic polymers, polylactic acid (PLA), polypropylene (PP), and polyamide (PA) have been extensively investigated for compounding with lignin particles. Additionally, epoxy resin/lignin composites have emerged as highly desirable blends in the field of thermoset polymers. Similarly, driven by concerns related to human health and the environment, numerous studies have been conducted on substituting carbon black (CB) with lignin particles in rubber-based composites.

### 7.1. Lignin integration with polylactic acid

**Fig. 15:**
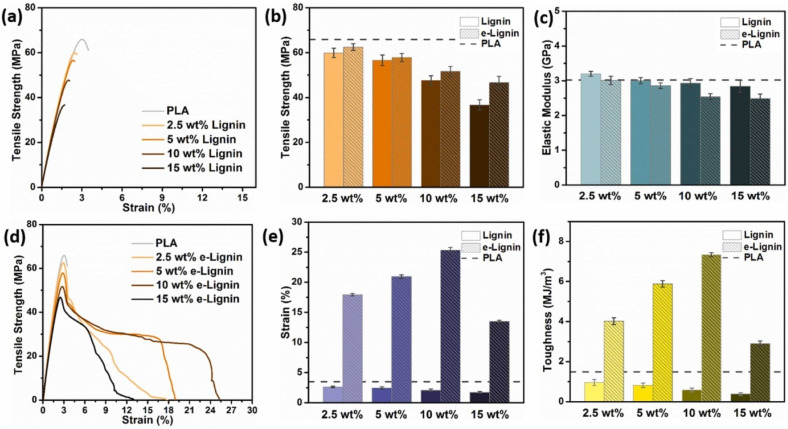
Stress–strain curves of (a) PLA/lignin composites and (d) PLA/e-Lignin composites made by injection molding. (b) Tensile strength, (c) modulus, (e) strain, and (f) toughness comparison of PLA/lignin composites and PLA/e-Lignin composites, reproduced with permission from Ding, 2023, Copyright (2023) Elsevier.^146^

In the recent consumption of synthetic polymers, significant attention has been raised to developing biobased and biodegradable polymers. These polymers are commonly derived from biobased resources and can be biodegradable and/or compostable. One of the well-established biobased polymers, a product of agricultural bioprocessing, is polylactic acid (PLA). PLA possesses numerous advantages, including biocompatibility, biodegradability, renewability, easy processability, and high strength; thus, it has found uses in automotive parts (floor mats, panels, and covers) and biomedical fields. However, a relatively high cost, as well as brittleness, poor heat resistance, and low crystallization, are some of the main drawbacks of PLA, limiting its broader application. Compounding PLA with fillers is a pathway to enhancing the aforementioned drawbacks. For instance, Ou *et al.*^145^ produced biocomposites of PLA and lignin particles as a novel reactive compatibilization using epoxidized natural rubber (ENR) to improve the interfacial adhesion and compatibility between lignin particles and PLA. They found that a thin layer of ENR covered lignin particles, resulting in excellent compatibility, attributed to the interfacial reaction between ENR and lignin particles. The improved compatibility significantly enhanced the mechanical properties of the PLA matrix. Namely, the tensile strength and elongation at break were respectively improved by 15% and 77%, in PLA containing 20 wt% lignin and only 1 wt% ENR. Likewise, ductile, printable PLA/lignin composites were devised through interface engineering.^146^ To this aim, lignin was incorporated into the PLA matrix through a filament extrusion process. The impact of lignin on the composite and their mechanical performance (PLA/lignin composite containing 10 wt% modified lignin, e-lignin) showed positive impacts on the toughness and impact energy, which was improved 3-fold relative to the pure PLA (toughness and impact energy was increased to 3.84 MJ m^−3^ and 6.36 kJ m^−2^, respectively). Fig. 15 presents the mechanical performance of the developed composites after blending PLA with different concentrations of plain lignin and e-lignin.

Similarly, Long *et al.*^147^ developed lignin nanospheres/PLA composites for fused deposition modeling (FDM). The lignin nanospheres, their low cost, good dispersibility, and high specific surface area have positive impacts on PLA/lignin composites to fabricate FDM filaments with uniform diameters. Consequently, some 3D objects were printed with high resolution and precise size. An optimum lignin addition improved the flexural strength, flexural modulus, and tensile strength of PLA/lignin composites. For instance, after compounding PLA with 0.5 wt% lignin, the flexural strength, the flexural modulus, and the tensile strength were 103 MPa, 2158 MPa, and 59.6 MPa, respectively (131%, 152%, and 56% improvement in the mentioned mechanical properties of the PLA matrix). These improvements were attributed to the effect of hydrogen bonding, hydrophobic effect, and π–π interactions involving lignin molecules. Zhang *et al.*^148^ developed PLA/lignocellulose nanofiber composites as 3D printable filaments of different lignin concentrations, from 0.2 to 30 wt%. They showed remarkable improvement in the PLA's flexural properties. Namely, in the presence of 10 wt% lignin, the composite presented the highest flexural strength, 234.5 MPa, 153% higher than pure PLA. This enhancement was attributed to the homogenous and even distribution of the reinforcing phase, lignin, in the PLA matrix. However, excess lignin impaired the mechanical properties, explained by the self-aggregation of the particles inside the matrix.

To produce a biodegradable PLA composite with high ductility and strength, lignin-containing cellulose nanofibrils (LCNF) were integrated into a PLA matrix.^149^ Lignin particles were used as modifiers to enhance the compatibility and the interfacial interaction between LCNF and PLA matrix through hydrogen bonding and van der Waals interactions. As a result, the mechanical properties were improved. For instance, a more than 260% increase was observed in the tensile strength of the composite compared to the neat PLA. Furthermore, the tensile modulus jumped from 1.7 GPa in pure PLA to 10.3 GPa in the composite. More importantly, the strain at break exceeded that of pure PLA film, and up to 400% improvement was reported. Anugwom *et al.*^150^ used lignin in biobased PLA/wood composites. By incorporating esterified lignin particles, it was possible to achieve a significant improvement in the tensile strength and tensile modulus of PLA/wood composites.

### 7.2. Lignin integration with polypropylene

Polypropylene (PP) is a widely used commodity polymer that presents numerous advantages, including low density, affordability, and good stiffness and strength. The inherent carbon footprint of PP can be significantly altered by incorporating fillers, fibers, or other polymers. For instance, blending PP with these materials can lead to improvements in strength and other properties. In this area, several studies have reported substantial enhancements through the incorporation of lignin-based materials.^151^

**Fig. 16:**
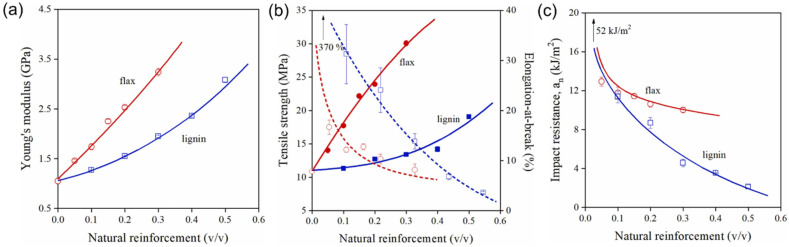
Different mechanical properties of PP composites blended with various concentrations of flax (○) and lignin (□), reproduced with permission from Pregi, 2022, Copyright (2022) Elsevier.^154^

Pregi *et al.*^152^ investigated the synergistic effect between flax and lignin on the properties of PP. Flax content of 0, 10, 20, and 30 vol% was tested at lignin concentrations varied between 0 and 50 vol% in a twin-screw compounder used to homogenize them for feeding into an injection molding device to prepare tensile coupons. An increase from 10 to 20 MPa in the tensile strength was measured upon the addition of lignin, and at the same time, the stiffness was enhanced from 1 to 2.5 GPa. However, quite a drastic reduction in impact strength was observed upon increasing the lignin content. Peng *et al.*^153^ blended PP with high lignin-content cellulose particles (HLCP) and reported 25.3% and 41.5% respective improvements in the tensile strength and modulus of the composites compared to neat PP. Similarly, composites were prepared from PP, lignin, and flax fibers for structural applications.^154^ To this goal, the focus of the study was mainly on mechanical strengthening, including stiffness and impact resistance. The effect of both lignin particles and flax fibers on the mechanical properties is presented in Fig. 16. Both components enhanced the tensile modulus of the polymer; however, for flax, the reinforcing effect was more substantial, thanks to its anisotropic nature. For instance, the tensile modulus of PP increased from 1.0 to 2.7 GPa after compounding with 50% (v/v) lignin particles; however, up to 3 GPa stiffness was reported by blending just 30% (v/v) flax fiber. Like stiffness, the reinforcing effect of flax exceeded by far that of lignin. Namely, in the presence of lignin, the tensile strength improved from 10 MPa to 18 MPa, while flax fibers caused a subsequent tensile strength of 30 MPa. On the other hand, stretchability decreased considerably after compounding PP with both additives. Finally, both components, *i.e.*, lignin and flax fiber, reduced fracture strength, where the presence of lignin depressed the impact resistance more remarkably than flax fibers.

The tensile and flexural strength and modulus of PP composites containing lignin and semi-bleached cellulose fibers were thoroughly investigated by Gadioli *et al.*^155^ An improvement in the mechanical properties of PP was observed. However, the improvement was more pronounced after the incorporation of lignin particles. In particular, 70% and 63% improvements were reported for the tensile and flexural strengths when the bleached fibers were incorporated in the PP matrix, confirming their reinforcing effect (83% and 73% in the composites containing the highest lignin content and semi-bleached fibers, respectively). The tensile and flexural moduli presented a similar trend. Specifically, tensile and flexural moduli improved proportionally to the lignin concentration in the fibers, which was attributed to the higher rigidity of the lignin-containing fibers and the positive impact of lignin on better fiber/matrix adhesion. Finally, lignin was studied as an additive in PP/coir composites to investigate its possible impact on thermal and mechanical properties.^156^ Different compositions of PP/coir/lignin composites were prepared, and up to 10 wt% lignin incorporation did not remarkably change the tensile strength of the composite. In contrast, considerable variability in elongation was reported.

### 7.3. Lignin in polyamide composites

Polyamides (PA), also known as nylons, are a well-known family of engineering plastics used in different applications, such as textiles, automobile parts, carpets, fishing lines, and packaging. These diverse applications benefit from the outstanding mechanical properties of PLA, excellent resistance against many solvents, high stiffness-toughness balance, and good thermal stability. Different types of PAs, PA6, and PA66, have been produced from petroleum-based monomers; bio-based PAs have also been developed with examples such as PA11, PA1010, and PA1012. Blending PAs with bio-based fillers has been considered recently as a green strategy not only to enhance the bio-content of PAs but also to improve the properties of PAs.

Baniasadi *et al.*^157^ grafted *n*-octadecyl isocyanate molecules on the surface of lignin to make it compatible with a polyamide matrix in the melt compounding process. They monitored different properties of the developed polyamide/compatibilized-lignin composites, including the mechanical properties, and realized improvements in stiffness, while the tensile strength and elongation at the break did not change dramatically after compounding the matrix with up to 40 wt% lignin. Furthermore, Ajdary *et al.*^158^ blended lignin particles with a PA12 matrix and developed 3D structures *via* a selective laser sintering technique. They used a fixed ratio of 60/40 for PA12 and lignin and examined the effect of printing orientations, including flat, flipped (90°), and vertical, on the mechanical properties of the composites. All printed samples showed an improvement in the tensile modulus, with the greatest improvement for the sample printed with a 90° orientation.

In another study, Muthuraj *et al.*^159^ pyrolyzed a composite containing hydroxypropyl-modified lignin and a biobased polyamide (PA1010) to develop new bio-based carbon filaments. Ethylene-methyl acrylate-glycidyl methacrylate and ethylene-acrylic ester-maleic anhydride (MA) were used as compatibilizers through a reactive extruder to improve the properties of the blends. Then, using a continuous melt-spinning process, they produced some filaments and then carbonized them into carbon fibers. For non-compatibilized and 2% MA compatibilized blends, tensile stress values of 192 ± 77 and 159 ± 95 MPa and moduli of 16.2 and 13.9 GPa were reported, respectively. Likewise, Sallem-Idrissi *et al.*^160^ developed fully bio-based compounds of lignin/PA11 using direct extrusion technology. No chemical pre or *in situ* modifications or physical pretreatments were employed. The authors thoroughly studied the mechanical and thermal properties of the blends and reported a slight reduction in the elongation at break after blending PA11 with 12.5% lignin particles. Excellent miscibility of lignin and PA11 matrix and the plasticization property of lignin were introduced as the main reasons for such small ductility reduction. However, a significant decrease was reported after blending PA11 with 25% lignin, which was attributed to restricted molecular mobility induced by lignin particles. This restriction resulted in a lack of deformability of the polymer within the interphase close to the particle. This composite, on the other hand, presented higher yield stress and tensile modulus compared to the neat PA11 matrix. Other reports on PA/lignin blends are available in the literature.^161,162^

### 7.4. Lignin integration into epoxy resin composites

Epoxy resin is widely employed in electronics, coatings, and various industries due to its remarkable mechanical properties and thermal stability. In epoxy composites, lignin offers considerable application potential. Lignin's functional groups facilitate strong interactions with the epoxy resin during the curing process, resulting in the formation of rigid three-dimensional network structures. Consequently, lignin serves as a component that enhances the properties of epoxy resin composites.

**Fig. 17:**
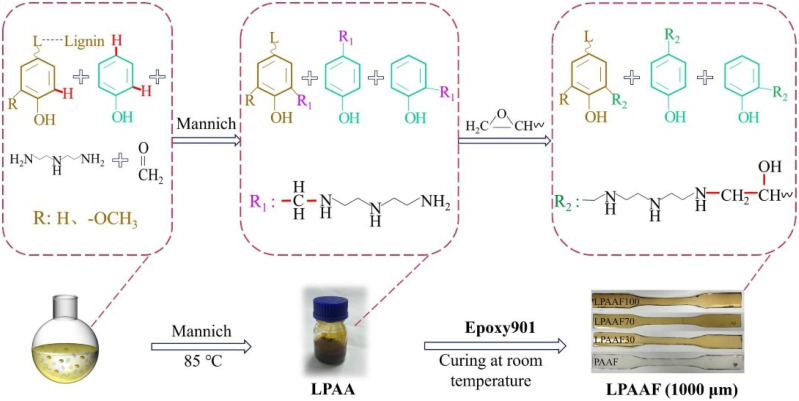
Schematic illustration of black liquor lignin-based phenolic amine synthesis and epoxy composite preparation, reproduced with permission from Li, 2023, Copyright (2023) Elsevier.^163^

Li *et al.*^163^ synthesized a black liquor lignin-based phenolic amine *via* the Mannich reaction and then utilized it to develop composite films from epoxy resins. The preparation process is schematically presented in Fig. 17. The highest toughness and the lowest tensile strength belonged to the sample without lignin, while the tensile strength improved upon increasing the liquor lignin loading. Specifically, after blending 70 wt% black liquor lignin, the composite provided tensile strength, *i.e.*, 30 MPa. This improvement was attributed to the reinforcement of inorganic chemicals in black liquor lignin and the rigid benzene ring structure of lignin.

Similarly, industrial alkali lignin was recently utilized to fabricate environmentally friendly bio-based epoxy thermosetting materials.^164^ The lignin-based epoxy resin was synthesized through fractionation and modification to adjust the properties of epoxy resin. To this aim, lignin was epoxidized and then cross-linked with various proportions of bisphenol A diglycidyl ether to fabricate thermosetting epoxies. It was approved that the cured resin possessed enhanced tensile strength, *e.g.*, 4.6 MPa, and elongation, *e.g.*, 315.5%, compared with the common polymer. Furthermore, the development and characterization of bioinspired mono-component lignin endowing epoxy resin were reported by Liu *et al.*^165^ Through electrostatic interaction, lignin was assembled with melamine and amino trimethyl phosphonic acid to synthesize a novel lignin-based flame retardant (LMA). LMA was then chemically incorporated into an epoxy resin in various proportions. The ring-opening reaction between the hydroxyl group in LMA and epoxy resin resulted in an excellent interfacial strength between the components. Thereby, impact strength, the tensile modulus, and tensile strength were enhanced by 53.7%, 25.7%, and 17.9%, respectively. Likewise, lignin-based epoxy resins were fabricated by mixing a conventional bisphenol A-based epoxy resin with depolymerized kraft/organosolv lignin. The lignin concentrations in the fabricated bio-based epoxy systems varied from 25 to 100 wt%. Various properties of the developed composites, including mechanical properties, were investigated. It was indicated that both the flexural strengths and the tensile strength of the composite with a pure lignin-based epoxy resin were significantly lower than those of the sample with the conventional bisphenol A-based epoxy; nevertheless, the flexural modulus or elastic modulus of the composite with pure lignin-based epoxy resins were all comparable.^166^

### 7.5. Lignin integration into rubber-based composites

In recent years, there has been a notable research focus on exploring natural fillers as substitutes for oil-based materials in the field of rubber composites. CB has traditionally been used as a reinforcing filler in rubber products to enhance mechanical properties and improve resistance to abrasion and fatigue. However, concerns about the adverse effects of CB on human health and the environment have spurred efforts to develop greener alternatives. Lignin, with its unique structure comprising poly-aromatic constituents, low average molecular weight, abundant renewable supply, environmental friendliness, and inherent reinforcing capabilities, has emerged as a promising green and sustainable alternative to CB.

Recently, Qiu *et al.*^167^ designed a lignin/silicon dioxide (SiO_2_) nano-hybrid, *i.e.*, DLSi, and investigated the reinforcing effect of DLSi on natural rubber (NR). Different formulations, including CB-filled natural rubber (NR-CB), were prepared. It was confirmed that the NR-DLSi composite presented comparable mechanical properties, *e.g.*, tensile strength, stress at the strain of 100%, and ultimate strain, to NR-CB. Furthermore, Mohamad Aini *et al.*^168^ incorporated CB/lignin into NR/polybutadiene rubber (BR). Liquid butadiene rubber (LBR-352) and epoxidized natural rubber with 50 mol% of epoxide group (ENR-50) were employed as compatibilizers, and their possible impacts on different properties of the developed composites, including the mechanical performance, were investigated and discussed. The LBR compatibilizer provided insufficient interaction between filler and rubber; thereby, the poorest tensile properties were reported. However, significant improvement and reinforcement were reported for ENR compatibilizer due to the reaction of the epoxy group of compatibilizer with the hydroxyl group of lignin and making rigid lignin-ENR networks. As a result, upon increasing ENR loading up to 10 phr (parts per hundred of rubber) the tensile strength and modulus at 100% and 300% strain were increased.

Similarly, the CB/lignin hybrid fillers were incorporated into a NR matrix, and a comparative study was performed to investigate their possible reinforcing impact.^169^ The NR/CB/lignin vulcanizates possessed a minor variation in tensile strength, while with the addition of less than 30 phr of lignin, the elongation at break gradually increased. Excellent dispersion of CB into the NR matrix, the high filler-rubber interaction at the interface, and the strain-induced crystallization behavior of the NR matrix were introduced as the main factors for the observed mechanical properties after blending NR with the hybrid filler particles. Moreover, compared to the lignin-free sample, it was realized that in NR containing 40 and 50 phr lignin particles, the tensile strength was reduced by 14.2% and 39%, respectively. Likewise, the modulus at 100% elongation and the modulus at 300% elongation were reduced after the incorporation of lignin. Poor filler dispersion in the NR matrix at high lignin contents and the decrease in crosslinking densities of NR, leading to inefficient load transfer at the interfaces, were proposed as the main reasons for a decrease in modulus values. Overall, it was concluded that by adjusting the vulcanization agent in the formulation, the tensile properties of the CB/lignin-filled rubber composites could be improved to fulfill the criteria of practical applications.

**Fig. 18:**
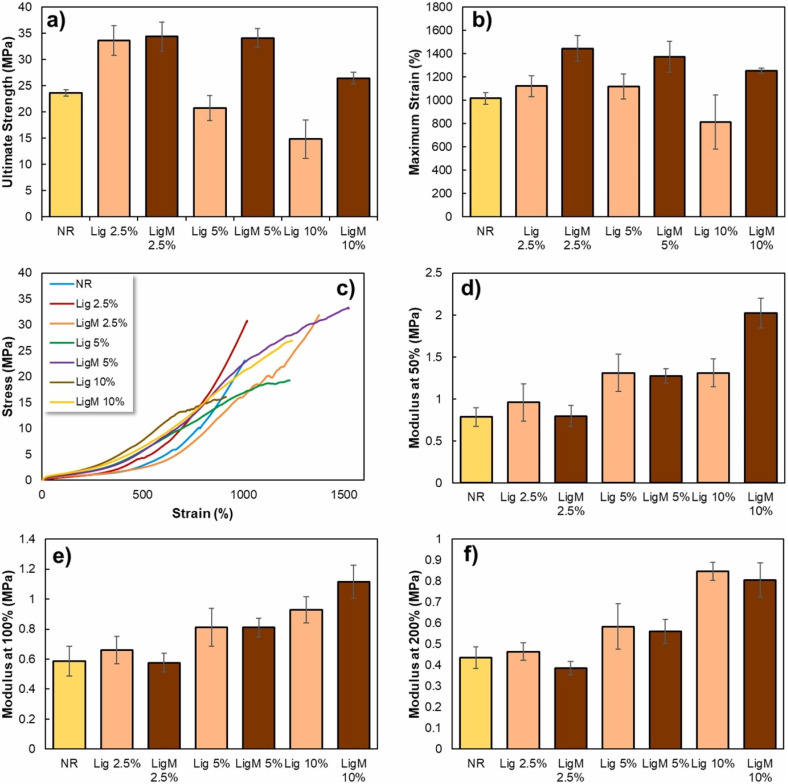
Tensile analysis of baseline natural rubber and its composites with lignin and modified lignin at various concentrations, (a) Ultimate strength, (b) Elongation at break, (c) Stress–strain plots, (d) Elastic modulus at 50%, (e) Elastic modulus at 100% and (f) Elastic modulus at 200%, reproduced with permission from Shorey, 2021, Copyright (2021) Elsevier.^170^

In another research work performed on lignin/rubber composites, Shorey *et al.*^170^ hydrophobized lignin particles and then blended them with a rubber matrix. They successfully hydrophobized kraft lignin by developing a novel silylation reaction. Thereafter, they dispersed the silylated kraft lignin into a natural rubber matrix and examined chemically modified lignin's effect on rubber composites’ mechanical properties. The results are summarized in Fig. 18, which shows that the NR matrix's tensile strength was significantly enhanced by adding 5 wt.% silylated lignin. Namely, compared to the unfilled NR, they observed a 44.4% increase in tensile strength. Further loading of silylated lignin into the NR matrix decreased the tensile strength attributed to the lignin particle aggregation and poor lignin-NR interactions. Furthermore, upon adding both unmodified and modified lignin, the elongation at break dropped considerably, probably due to the incorporation of rigid lignin moiety in the matrix as well as the strong filler–filler interactions, which restricted the mobility of the elastomeric moieties. Finally, lignin incorporation in castor oil-based elastomers has set new limits in rheology and cushioning properties.^171^

In conclusion, the incorporation of lignin into polymer composites has shown promising results in enhancing mechanical properties, and sustainability, and reducing the carbon footprint of various polymers. Lignin, a bio-based filler, is particularly attractive due to its unique chemical structure, availability, reinforcement capabilities, biodegradability, and renewability. The compatibility between lignin and polymers, especially hydrophobic ones, poses a significant challenge, leading to the development of various modification methods.

In the context of specific polymers, such as polylactic acid (PLA), polypropylene (PP), and polyamide (PA), the addition of lignin has demonstrated improvements in mechanical properties, providing solutions to some of the inherent drawbacks of these polymers. For PLA, in particular, the brittleness and poor heat resistance were addressed by compounding with lignin, leading to increased tensile strength, elongation at break, and toughness. Similarly, lignin incorporation into PP and PA resulted in enhanced tensile and flexural strength, though trade-offs were observed in impact resistance and stretchability. In epoxy resin composites, lignin has been utilized to create rigid three-dimensional network structures, capitalizing on its compatibility and functional groups that facilitate strong interactions during the curing process. The resulting composites exhibited improved mechanical properties, such as tensile strength and toughness. Furthermore, lignin has emerged as a green alternative to carbon black in rubber composites, contributing to comparable mechanical properties while addressing health and environmental concerns associated with carbon black.

However, challenges and issues persist, including the need for further advancements in lignin modification techniques to enhance compatibility with a wider range of polymers. Additionally, the optimization of formulations, especially in terms of lignin content, remains a critical aspect, as excessive lignin can lead to particle aggregation and a decrease in mechanical properties. Moreover, the economic viability of these lignin-based composites, considering the cost of lignin extraction and modification, requires attention for broader industrial adoption.

Looking to the future, research should focus on refining the processing methods, exploring novel lignin sources, and addressing issues related to scalability and cost-effectiveness. Collaborative efforts between academia and industry will be essential to bridge the gap between laboratory-scale successes and large-scale production. As lignin-based polymer composites continue to evolve, they hold great promise in contributing to sustainable and eco-friendly material solutions across various industries.

## 8. Summary

In recent times, there has been a growing demand for biodegradable, eco-friendly, and sustainable alternatives. Notably, lignin has gained significant attention as a material that is both environmentally friendly and economically beneficial. This review focused on exploring the diverse functional applications of lignin materials, ranging from adhesive and antibacterial agents to rheology modifiers and reinforcing fillers. The review examined and discussed the latest research conducted in recent years on the utilization of lignin in emerging composite materials. Specifically, it explored the potential of lignin as a substitute for phenol in adhesive applications. The conclusion drawn is that lignin in adhesives offers an eco-friendly solution for environmental concerns and cost reduction in the wood composite industry. The shift from petroleum-derived adhesives, causing environmental and health issues, is addressed by lignin, an affordable and renewable alternative to phenol. Lignin-based adhesives, both formaldehyde and formaldehyde-free, undergo modifications like ionic liquid treatment and glyoxalation to enhance reactivity. Challenges persist, such as color and curing rate in lignin formaldehyde resins. Lignin-furfural and lignin-polyurethane adhesives exemplify lignin's versatility in creating formaldehyde-free options with biodegradability. Alternative approaches like lignin–tannin and lignin-soy protein adhesives showcase diverse strategies, with ongoing efforts to optimize formulations.

Furthermore, it can be concluded the incorporation of lignin and lignin-based materials into polymer composites offers a versatile strategy to enhance their thermal properties across various applications. The intrinsic aromatic structure of lignin contributes to the remarkable thermal stability and UV resistance observed in these composites. Lignin, particularly in the form of nanoparticles, serves as an effective thermal stabilizer by absorbing and dissipating heat, promoting better dispersion within the polymer matrix, and acting as nucleation sites for crystallization. These mechanisms collectively lead to improvements in thermal conductivity, stability, and fire resistance.

Our review exhibited how lignin was suitably modified and appropriately utilized for making materials that have improved barrier properties. As we know, lignin is a large molecule containing various functional groups, that are considered beneficial from the synthetic viewpoint, but these functional groups arise from different extraction and treatment processes that also result in heterogeneity and variability in the quality of lignin. The source of lignin is also a variability parameter that can influence the functional efficacy of a composite when applied as a flame retardant, for example. Thus, further research on physicochemical properties, material compatibility, and surface chemistry related to lignin and nano lignin is essential. The extent and means of lignin extraction and modification (for example, halogenated agents for inducing hydrophobicity or flame retardancy that ultimately makes the material non-biodegradable) should be taken care of from an environmental viewpoint. Nano lignin when modified or blended with various other bio-based matrices has the potential for improved barrier properties, that also need evaluation of environmental, social, and economic sustainability to take these materials from lab to industrial scale.

Additionally, the presence of lignin is observed to increase storage and loss moduli, relaxation time, and melt viscosity in different polymer matrices. Several studies point to the shear-thinning effect of lignin, influencing the behavior of polymer blends and preventing crystal reaggregation. The addition of lignin in various composite systems, such as ABS and PLA, affects the elastic characteristics and dynamic responses.

Finally, the review shed light on the role of lignin as a reinforcement material for improving the mechanical properties of polymers. Different polymer matrices blended with lignin materials were studied, and it is pointed out that the final properties of the developed composites with lignin particles primarily depend on the compatibility between lignin and the polymer matrix. In many research works in this field, various compatibilization approaches have been employed to enhance the compatibility between phases and, consequently, improve the mechanical performance of the final composite. In conclusion, this review paper highlighted the growing interest in lignin as a versatile and sustainable material with various functional applications in composite materials and underscored lignin's promising role in advancing the development of biodegradable, eco-friendly, and sustainable materials.

## 9. Future and outlook

Lignin-based composites have already demonstrated their potential across several industries, including construction, automotive, packaging, and coatings. However, there is still significant untapped potential to explore regarding lignin's unique properties and its compatibility with other materials. Advancements in lignin modification hold the key to producing tailor-made lignin derivatives with specific properties and functionalities, opening up a broader range of applications. For instance, lignin-containing cellulose nanofiber exhibited a stabilizing effect on Pickering emulsions.^172^ In another story regarding paper coating, nanocellulose containing lignin (produced from unbleached pulp) will provide the opportunity in the future to use unbleached pulp in the paper industry, which will be revolutionary.^173^ Lignin, further, can be utilized in novel fiber composites with cellulose nanocrystals and polyvinyl alcohol using electrospinning. Such fiber mat composites demonstrated mesmerizing morphological and thermal characteristics and opened the door to shaping new functional materials.^174^ Similarly, a system from the wet spinning of lignin and cellulose nanofiber can be altered to high-value carbon fibers *via* carbonization.^175^ Furthermore, high-value structures such as multiwall carbon structures, metal-phenolic networks, and supercapacitance electrodes can be achieved based on lignin nano derivatives.^176^ Regarding the concept of novel multiphase systems, carboxymethylated lignin can be utilized to produce foam with interesting stability and develop web-based products as well as stabilizing effects on fuel emulsions.^177^ Lignin, eventually, showed to have potential in the introduction of membranes and films into composites. The combination of lignin (chemically modified and unmodified) and other materials provided the opportunity to produce antioxidant membranes, carbon membranes, and strong composites.^178^

To wrap up, all the examples given in this review shed light on the fact that lignin and its derivatives contribute to reducing dependence on non-renewable resources, mitigate the environmental impact associated with conventional plastics and composites, and will have a bright future in the coming era. However, it is essential to conduct comprehensive life cycle assessments to evaluate the greenhouse gas emissions and overall environmental impact of lignin-based composites. This holistic approach ensures that the environmental benefits of utilizing lignin are accurately assessed and guides the responsible development and application of lignin-based materials.

## Abbreviations

ABS: Acrylonitrile butadiene styrene
BPA: Bisphenol A
CB: Carbon black
CNC: Cellulose nano crystals
CNF: Cellulose nano fibers/fibrils
ENR: Epoxidized natural rubber
FR: Fire retardancy
GMA: Glycidyl methacrylate
HDPE: High-density polyethylene
HRR: Heat release rate
KL: Kraft lignin
LCNF: Lignin-containing cellulose nanofibrils
LFSol: Soluble lignin fraction
LNM: Lignin nano micelles
LNP: Lignin nanoparticles
LOI: Limiting oxygen index
LPF: Lignin–phenol–formaldehyde
LS: Lignosulfonate
MA: Maleic anhydride
NR: Natural rubber
PA: Polyamide
PAN: Polyacrylonitrile
PBAT: Poly(butylene adipate-*co*-terephthalate)
PE: Polyethylene
PF: Phenol formaldehyde
PGM: Phosphated lignocellulose nanofibrils, gelatin, and MXene
PLA: Poly-lactic acid
PMMA: Polymethyl methacrylate
POSS: Polyhedral oligomeric silsesquioxane
PP: Polypropylene
PVA: Poly (vinyl alcohol)
TGA: Differential scanning calorimetry
UF: Urea formaldehyde
UV: Ultraviolet

## Author contributions

Mahyar Fazeli: conceptualization, supervision, investigation, visualization, writing – original draft, writing – review & editing. Sritama Mukherjee: visualization, investigation, writing – original draft, writing – review & editing. Hossein Baniasadi: visualization, investigation, writing – original draft, writing – review & editing. Roozbeh Abidnejad: visualization, investigation, writing – original draft, writing – review & editing. Muhammad Mujtaba: visualization, investigation, writing – original draft, writing – review & editing. Juha Lipponen: funding acquisition, supervision, writing – review & editing. Jukka Seppälä: funding acquisition, supervision, writing – review & editing. Orlando J. Rojas: funding acquisition, supervision, writing – review & editing.

## Conflicts of interest

The authors confirm that they are not affiliated with or involved in any organization or entity that has a financial or non-financial interest in the subject matter or materials covered in this article.

## Supplementary Material
